# The development of a Self-Rated ICF-based questionnaire (HEAR-COMMAND Tool) to evaluate Hearing, Communication, and Conversation disability: Multinational experts’ and patients’ perspectives

**DOI:** 10.3389/fresc.2022.1005525

**Published:** 2022-11-14

**Authors:** Tahereh Afghah, Razan Alfakir, Markus Meis, Lisette van Leeuwen, Sophia E. Kramer, Mahmoud Hammady, Mostafa Youssif, Kirsten C. Wagener

**Affiliations:** ^1^ Hörzentrum Oldenburg gGmbH, Germany and Cluster of Excellence Hearing4all, Oldenburg, Germany; ^2^ Department of Speech-Language and Hearing Sciences, Auburn University, Auburn, AL, United States; ^3^ Department of Otolaryngology, Head and Neck Surgery, Amsterdam University Medical Center, Amsterdam, Netherlands; ^4^ Department of Otolaryngology, Head and Neck Surgery, Audiovestibular Medicine Division, Sohag University Hospital, Sohag, Egypt

**Keywords:** International Classification of Functioning disability and health, hearing loss questionnaire, hearing impairment, ICF core sets for hearing loss, communication disability, outcome measure

## Abstract

**Objective:**

An instrument that facilitates the advancement of hearing healthcare delivery from a biomedical model to a biopsychosocial one that underpins the International Classification of Functioning, Disability, and Health framework (ICF) brief and comprehensive Core Sets for Hearing Loss (CSHL) is currently unavailable. The objective is to describe the process of developing and validating a new questionnaire named the *HEAR-COMMAND Tool* created by transferring the ICF CSHL into a theory-supported, practically manageable concept.

**Design:**

A team from Germany, the USA, the Netherlands, and Egypt collaborated on development. The following ICF domains were considered; “Body Functions” (BF), “Activities and Participation” (AP), and “Environmental Factors” (EF). The development yielded English, German, and Arabic versions. A pilot validation study with a total of 109 respondents across three countries, Germany, Egypt, and the USA was conducted to revise the item terminology according to the feedback provided by the respondents.

**Results:**

The questionnaire included a total of 120 items. Ninety items were designed to collect information on the functioning and 30 items inquiring about demographic information, hearing status, and Personal Factors. Except for the “Body Structures” (BS) domain, all the categories of the brief ICF CSHL were covered (a total of 85% of the categories). Moreover, the items covered 44% of the comprehensive ICF CSHL categories including 73% of BF, 55% of AP, and 27% of EF domains. Overall, the terminology of 24 ICF-based items was revised based on the qualitative analysis of the respondents' feedback to further clarify the items that were found tod be unclear or misleading. The tool highlighted the broad connection of HL with bodily health and contextual factors.

**Conclusions:**

The *HEAR-COMMAND Tool* was developed based on the ICF CSHL and from multinational experts' and patients' perspectives with the aim to improve the execution of audiological services, treatment, and rehabilitation for adult patients with HL. Additional validation of the tool is ongoing. The next step would be to pair the tool with BS categories since it was excluded from the tool and determine its effectiveness in guiding hearing health care practitioners to holistically classify categories influencing hearing, communication, and conversation disability.

## 1. Introduction

Current healthcare systems in general and hearing healthcare systems in specific, nationally and internationally, are increasingly challenged to meet the needs of a growing number of patients diagnosed with Hearing Loss (HL) (1). The impact of HL on an individual's functioning is highly dependent on the etiology and pathological process, the magnitude of the loss, and the individual's lifestyle, communication needs, and specific environment (2). This highlights the need for a personalized approach to hearing healthcare that goes along the lines of a globally accepted evaluation standard.

HL is a potentially disabling health condition and can have a substantial impact on an individual's participation, psychosocial interaction, and quality of life (3). Due to this complex impact on an individual's functioning, the burden of HL from a population perspective is potentially hard to detect and quantify even in well-designed prospective studies. It has been suggested that the diversity of the available instruments in terms of content, response options, and administration hinders the comparability of studies and the performance of meta-analyses (4, 5). Hence, converging the measures applied in the available hearing health care is essential for identifying key indicators for the quality of audiologic hearing rehabilitation (AR) procedures.

Throughout the AR procedures (primarily hearing technology and communication strategies), it is expected that the hearing aid would improve the patients’ auditory function and as a result, improve communication and conversation disability. Despite hearing aid benefits, communication and conversation difficulties persist. Communication requires thinking and the ability to convey the message *via* meaningful messages verbally or non-verbally. Conversation difficulty requires the individual's ability to initiate and maintain a dialogue with others. Communication and conversation difficulties of persons with HL and encountered by Hearing Healthcare Professionals (HHPs) can be divided broadly into impairments of mental functions, sensory functions, and speech and voice functions. All these impairments are highly influenced by personal factors (i.e., aging and/or comorbid processes and diseases related to the physiologic systems) and environmental factors (i.e., HHPs scope of practice, social attitude, and healthcare systems) (6). This in turn may aggregate the onset and progression of HL, risk for future disability, and health care utilization. Hence, many adults experience HL as a very disabling condition (7). Failure to assess broad aspects of hearing, communication, and conversation disability and offer an array of healthcare intervention options targeted to patients’ needs can be a major factor contributing to low AR outcomes such as hearing aid uptake, use, benefits, and satisfaction (8). The dominant model of HL today is biomedical, and this leaves no room within its framework for the broad aspects of hearing, communication, and conversation disability. Further, due to the workplace (e.g., lack of time in appointments and support from the workplace), capability (e.g., lack of knowledge, skills, confidence in recognizing psychological symptoms, and comfort in discussing mental health), education (e.g., insufficient training about mental health and illnesses), and recognition (e.g., lack of outcome measure that captures what matters to people living with HL and allows HHPs to go beyond the traditional standards of hearing healthcare) HHPs are not sufficiently addressing broad aspects of hearing, communication, and conversation disability or consider them when planning management (6–8).

The first pivotal need to define a method to appraise the efficacy of AR procedures is to design an assessment method that is implemented according to a broad standard. The required method must consider hearing impairment and all the potential effects of that on a patient's life. At the same time, the method has to be globally accepted to allow for universal usage. To implement such a methodology, applying the World Health Organization's International Classification of Functioning Disability and Health (ICF) framework (9) was found to be an ideal design foundation. The ICF is an internationally accepted standard and its applicability to assess HL and AR has been advocated for many years (10–12). Functioning refers to positive aspects of Body Structure (BS) and Body Functions (BF), Activities and Participation (AP), while disability is an umbrella term for impairments, activity limitations, and participation restrictions. In the ICF, both aspects can be influenced by a health condition(s) and or contextual factors, including Personal Factors (PF) and Environmental Factors (EF).

To address HL from both a biomedical (medical/disease) and broad aspect of hearing, communication, and conversation disability, the comprehensive and brief ICF Core Sets for HL (CSHL) have been developed (13, 14). The comprehensive and brief ICF CSHL are the shortlists of categories selected from the generic ICF that are considered the most essential when assessing and reporting the functioning of persons with HL or in the context of specific healthcare or health-related setting (13–18). These shortlists were created to make the ICF more applicable regarding HL assessment for everyday use in hearing health care and research. This makes both ICF CSHL promising tools for addressing the impact of HL or required interventions at the clinical, service, and public health levels.

Previous studies showed how the ICF CSHL could be operationalized into a self-assessment tool in AR clinical practice (19–25). These studies focused on developing a more specific content of the ICF CSHL to facilitate its semantic interoperability and implementation in audiological rehabilitation settings. Furthermore, the ICF has been applied widely in the literature to describe and differentiate the broad implications of HL and mental health on communication (23, 26–29). Case in point, in Alfakir & Holmes's study (23), the factor analysis of the questionnaire demonstrated a complex relationship between the auditory-related functions (speech understanding, listening, communication, and conversation) and mental-related functions (temperament and personality, attention, working memory, and emotion). Further, a recent study validates the ICF CSHL operationalized by the ICF-based questions from an international perspective (30). In Karlsson's study (30), the results revealed a six-factor solution focusing on issues related to communication, the social environment, participation in society, health care services, support, relationships, and emotions. However, in Karlsson's study, the ICF-based questions were developed as a questionnaire to enhance the quality of the data collection of the study rather than as a measurement tool. The primary outcome of these studies highlighted a need for developing a more specific content of the ICF CSHL to facilitate its semantic interoperability and implementation in AR settings.

The objective of this paper is to describe the process of developing a new ICF-based questionnaire named the *HEAR-COMMAND Tool*. The development of this questionnaire is part of a larger project aimed at providing the scientific basis for the assessment of an individual's disability due to HL and the hearing aid usage benefit with optimum ecological validity by developing rehabilitative measures that cover communication abilities as well as social interactions and participation. To achieve this, the two ICF CSHL were used as a basis for the development of these assessment methods.

## 2. Materials and methods

### 2.1. Developers

As the ICF is a worldwide accepted standard and is used internationally, having a team of experts from different countries was found remarkably propitious in the procedure of developing the *HEAR-COMMAND Tool*. Hence, an international team of experts with different scientific backgrounds gathered to build the questionnaire. The development team included experts from Germany, the United States, the Netherlands, and Egypt. The questionnaire design benefited from their expertise in audiology, medicine, psychology, neuropsychology, ICF applications, and questionnaire design.

### References

2.2.

Thirteen questionnaires focused on assessing impairment, health, and HL were partially used in the design. Table 1 illustrates these references along with the abbreviation of their titles used in this paper.

**Table 1:** Thirteen questionnaires focused on assessing impairment, health, and hearing loss that were partially used in the *HEAR-COMMAND Tool* development.

Questionnaire	Abbreviation	Developer (s)	Reference
Self-Assessments ICF Core Sets for Hearing Loss Questionnaire	Alfakir Questionnaire	Alfakir & Holmes 2017	(23)
An ICF-Based e-Intake Tool in Clinical Otology and Audiology Practice	van Leeuwen e-Intake Tool	van Leeuwen et al. 2020a	(24)
National Health And Nutrition Examination Survey-Audiometry	NHANES	United States Centers for Disease Control and Prevention (CDC) 2007–2008	(31)
World Health Organization (WHO) Disability Assessment Schedule	WHODAS 2.0	Üstün et al. and WHO 2010	(32)
World Health Survey Individual Questionnaire	WHS	WHO 2002	(33)
The Speech, Spatial and Qualities of hearing scale	SSQ	Gatehouse & Noble 2004	(34)
Dizziness Handicap Inventory	DHI	Jacobson et al. 1990	(35)
Self-Assessment of Communication	SAC	Schow & Nerbonne 1982	(36)
The ICF checklist Version 2.1a	ICF checklist	WHO 2007	(37)
Otology Questionnaire Amsterdam	OQUA	Bruinewoud et al. 2018	(38)
Hearing, Lifestyle and Health Questionnaire (Fragebogen Hören, Lebensgewohnheiten und Gesundheit)	HLHQ	Hörzentrum Oldenburg gGmbH, Gieseler et al. 2017	(39)
Ohrstrom Hearing Loss medical interview	OHL	Hörzentrum Oldenburg gGmbH, gathered in Afghah, et al. 2022	(40)
Amsterdam Inventory for Auditory Disability and Handicap	AIADH	Kramer et al. 1995	(41)

### 2.3. Design

The Consensus-based Standards for the selection of health Measurement Instruments (COSMIN) study design checklist was adopted in the development procedure (42). The *HEAR-COMMAND Tool* development procedure included the following steps.

#### 2.3.1. Selecting and formulating items

Throughout this manuscript, the questions of the *HEAR-COMMAND Tool* are referred to as “items”. The ICF-based items are referred to as H.n and the demographic information items are referred to as A.n where “n” represents the item number.

##### 2.3.1.1. ICF categories selection to be represented in the questionnaire (step 1)

The ICF categories are hierarchically organized in a stem-branch-leaf scheme using interlinked levels. The first-level categories (chapters) refer to general concepts and categories at a higher level are more detailed. General concepts are not suitable for operationalization into a questionnaire. To inquire about the disability and limitation degree, detailed questions with a unique and specified problematic or difficult condition/situation are required. Hence, only second and third-level items were selected to be represented in the questionnaire which describe a detailed concept.

Five sets of ICF categories were chosen for inclusion in the questionnaire. The *first* set of categories that were selected for inclusion in the questionnaire included all the (second-level) categories of the brief ICF CSHL (e.g., b126). The *second* selected set included the third-level categories of the comprehensive ICF CSHL that their corresponding second-level ICF categories are included in the brief ICF CSHL (e.g., b2301). The *third* selected set included the third-level ICF categories of the comprehensive ICF CSHL that corresponding second-level ICF categories are not included in the brief ICF CSHL (e.g., b1560). The *fourth* selected set included the second-level ICF categories of the comprehensive ICF CSHL that are not included in the brief ICF CSHL (e.g., d355). The *fifth* selected set consisted of the ICF categories neither included in the brief nor the comprehensive ICF CSHL, but still, the developers deemed fundamental for AR (e.g., b134).

##### 2.3.1.2. Item creation and terminology adjustment for the selected ICF categories (step 2)

First, a pool of items from existing questionnaires was created and linked to the selected ICF categories in step 1. Second, the terminology of the linked items was compared for each ICF category. As a result, three types of items were created; (1) Original items: The original item of one questionnaire was used as the primary source and it was slightly adapted according to the standard item formulation used in the *HEAR-COMMAND Tool* (the item formulation is explained in step 3). (2) Modified items: The original items of multiple questionnaires linked to a single ICF category were combined and modified accordingly and adapted to the *HEAR-COMMAND Tool* item formulation standard. (3) New items: For the selected categories in which the concept was not addressed in any of the references or the available questions were not usable for the purpose of this study, a new item was developed. In this case, the terminology of the item was adapted from the official description of the corresponding ICF category as formulated by the WHO. For instance, to create an item for the selected ICF category “Complex interpersonal interactions” (d720) with the description “*Maintaining and managing interactions with other people, in a contextually and socially appropriate manner, such as by regulating emotions and impulses, controlling verbal and physical aggression, acting independently in social interactions, and acting in accordance with social rules and conventions”*, the following item was created. H.61: “*Do you have difficulty with starting and continuing relationships in a socially appropriate manner (e.g., regulating emotions, controlling verbal and physical aggression)?*”. Table 2 illustrates the number of original, modified, and new items that were created for each ICF domain.

**Table 2:** The absolute number of new, modified or original items developed for each domain of the International Classification of Functioning, Disability and Health (ICF).

ICF domain	New	Modified	Original	Total
Body Functions	18	5	25	48
Body Structures	0	0	0	0
Activities and Participation	8	1	17	26
Environmental Factors	10	1	5	16
All domains	36	7	47	90

##### 2.3.1.3. Specific item formulation, scoring method, and experts' feedback (step 3)

The item formulation and the response options were synchronized with the ICF terminology and qualifiers (9, 37, 43,44). The items were shared with experts and adapted according to their suggestions. This feedback loop of modification and revision of the items was repeated five times. For all items, rules were drawn up to secure uniform formulations.

#### 2.3.2. Content evaluation

##### 2.3.2.1. Questionnaire beta version; experts' perspectives (step 4)

As the questionnaire was developed to be used internationally, evaluating it in more than one language and allowing for cross-cultural comparisons and adaptation were essential (45). Therefore, the developed items were translated into German and Arabic. The German version was translated at Hörzentrum Oldenburg gGmbH, Germany. The translation was performed by hearing specialists who are German native speakers and have English full professional proficiency. In case the translation of the applied terminology of an English item was not clear or meaningful in German, the wordings were reformulated in both languages to aim for the same meaning. After unifying the English and German versions, the initial version (i.e., beta version) of the questionnaire was created. Next, the beta version was translated from English to Arabic by native Arabic hearing specialists who have English full professional proficiency. The Arabic translation was then certified by the Sohag language and translation center, Sohag university, Egypt.

##### 2.3.2.2. Questionnaire revised version; patients' perspectives (step 5)

Up to this point, the defined terminology applied in the beta version was based on the experts' perspectives. It was essential to obtain the respondents' opinions regarding the questionnaire in general as well as on item-level. Therefore, the questionnaire was presented to 109 respondents in their native languages in Germany (*n* = 53), the USA (*n* = 26), and Egypt (*n* = 30). In Germany, the respondents were selected from a database of subjects with normal hearing and HL patients at Hörzentrum Oldenburg gGmbH who have previously signed up to participate in the studies of interest. In the USA, the respondents were selected from a database of patients visiting the Hearing and Speech Clinic at the Department of Speech-Language and Hearing Sciences, Auburn University. In Egypt, the respondents were selected from a database of patients visiting the Department of Otolaryngology, Head and Neck Surgery, Audiovestibular Medicine Division at Sohag University Hospital.

The respondents were asked to fill in the questionnaire and provide their feedback on the items either verbally or in writing. They were asked to identify the items that were found to be unclear, ambiguous, not meaningful, may potentially have more than one meaning, or difficult to grade.

## 3. Results

### 3.1. Selecting and formulating items

#### 3.1.1. ICF categories selection (corresponding to step 1)

Table 3 illustrates the ICF categories covered in the design of the *HEAR-COMMAND Tool* along with the item number and the references which were used to develop each. In this table, the inclusion of the categories of each ICF CSHL is demonstrated. The expanded version of this table that includes the terminology of each item along with the item type (new, modified, or original) can be found in the Supplementary material (1), Table 1.

**Table 3:** The International Classification of Functioning, Disability and Health (ICF) categories of the Core Set for Hearing Loss (CSHL) covered in the tool along with the item number and the references used for the item development.

Code	Category	Brief CSHL	Comprehensive CSHL	Extra categories	Item number	Reference
Body Functions						
b126	Temperament and personality functions	✓	✓	−	H.1	−
b134	Sleep functions	−	−	✓	H.2	(24, 33)
b140	Attention functions	✓	✓	−	H.3/H.4	(23, 32, 34, 35, 38)
b144	Memory functions	✓	✓	−	H.5/H.6	(23, 33)
b152	Emotional functions	✓	✓	−	H.7	(23, 33, 35, 38)
b1560	Auditory perception	−	✓	−	H.21/H.22/H.23/H.35/H.36/H.37	(23)
b167	Mental functions of language	−	✓	−	H.16/H.17	−
b210	Seeing functions	✓	✓	−	H.8/H.9	(32)
b2300	Sound detection	✓ (under b230)	✓	−	H.24/H.25	(41)
b2301	Sound discrimination	✓ (under b230)	✓	−	H.26/H.27/H.34	(34, 41)
b2302	Localisation of sound source	✓ (under b230)	✓	−	H.28/H.29/H.30/H.31/H.32	(34, 41)
b2303	Lateralization of sound	✓ (under b230)	−	✓	H.33	(34)
b2304	Speech discrimination	✓ (under b230)	✓	−	H.38/H.39/H.40	(34, 41)
b240	Sensations associated with hearing and vestibular functions	✓	✓	−	H.12/H.13/H.18/H.19/H.20	(23, 24, 31, 38)
b250	Taste function	−	−	✓	H.10	(24, 38)
b255	Smell function	−	−	✓	H.11	(24, 38)
b280	Sensation of pain	−	✓	−	H.14/H.15	(33)
b310	Voice functions	−	✓	−	H.41/H.42/H.43/H.44	−
b320	Articulation functions	−	✓	−	H.45/H.46	−
b330	Fluency and rhythm of speech functions	−	✓	−	H.47	−
Activities and Participation						
d110	Watching	−	✓	−	H.90	−
d115	Listening	✓	✓	−	H.74	(23)
d160	Focusing attention	−	✓	−	H.3/H.4	−
d220	Undertaking multiple tasks	−	✓	−	H.63/H.64/H.65/H.66	−
d240	Handling stress and other psychological demands	✓	✓	−	H.49	(24, 32)
d310	Communicating with—receiving—spoken messages	✓	✓	−	H.57	(23)
d330	Speaking	−	✓	−	H.48	−
d3500	Starting a conversation	✓ (under d350)	−	✓	Merged with H.67 and H.68	−
d3501	Sustaining a conversation	✓ (under d350)	−	✓	Merged with H.67 and H.68	
d3502	Ending a conversation	✓ (under d350)	−	✓	Merged with H.67 and H.68	
d3503	Conversing with one person	✓ (under d350)	✓	−	H.67/H.69/H.70/H.73	(34, 36, 41)
d3504	Conversing with many people	✓ (under d350)	✓	−	H.68/H.71	(23, 32, 36, 41)
d355	Discussion	−	✓	−	H.56	−
d360	Using communication devices and techniques	✓	✓	−	H.62/H.72	(41)
d710	Basic interpersonal interactions	−	✓	−	H.50	−
d720	Complex interpersonal interactions	−	✓	−	H.61	−
d730	Relating with strangers	−	✓	−	H.52	(32)
d740	Formal relationships	−	✓	−	H.53	−
d750	Informal social relationships	−	✓	−	H.51/H.54/H.55	(23, 32)
d760	Family relationships	✓	✓	−	H.58	(23, 24)
d820	School education	✓	✓	−	H.63/H.64/H.65/H.66	(32)
d830	Higher education	−	✓	−	H.63/H.64/H.65/H.66	
d850	Remunerative employment	✓	✓	−	H.63/H.64/H.65/H.66	
d855	Non—remunerative employment	−	✓	−	H.63/H.64/H.65/H.66	
d910	Community life	✓	✓	−	H.59	(32)
d920	Recreation and leisure	−	✓	−	H.60	−
Environmental Factors						
e125	Products and technology for communication	✓	✓	−	H.87/H.88/H.89/H.90	(23, 24)
e150	Design construction and building products and technology of buildings for public use	−	✓	−	H.81	−
e240	Light	−	✓	−	H.82	−
e2500	Sound intensity	✓ (under e250)	✓	−	H.83	−
e2501	Sound quality	✓ (under e250)	✓	−	H.84/H.85/H.86	(23, 36)
e310	Immediate family	✓	✓	−	H.77	(23, 24)
e320	Friends	−	✓	−	H.77	
e355	Health professionals	✓	✓	−	H.79	(24)
e410	Individual attitudes of immediate family members	✓	✓	−	H.76	−
e420	Individual attitude of friends	✓	✓	−	H.76	
e460	Societal attitudes	✓	✓	−	H.75	(24, 36)
e535	Communication services, systems and policies	−	✓	−	H.80	−
e580	Health services, systems and policies	✓	✓	−	H.78	−

The inclusion of the categories in each CSHL is shown by symbol ✓. The ICF-based items are referred to as H.n where “n” represents the item number.

Table 4 represents the absolute number of the categories of the two ICF CSHL that are reflected in the questionnaire along with the corresponding percentage concerning all of the ICF categories of that domain.

**Table 4:** The absolute number and the corresponding percentage of the covered categories of the International Classification of Functioning, Disability and Health (ICF) brief and comprehensive Core Sets for Hearing Loss in the *HEAR-COMMAND Tool*.

	Brief Core Set for Hearing Loss		Comprehensive Core Set for Hearing Loss	
ICF domain	*n*	%	*n*	%
Body Function	7	100	16	73
Body Structures	0	0	0	0
Activities and Participation	9	100	23	55
Environmental Factors	7	100	13	27
All domains	23	85	52	44

#### 3.1.2. Operationalization and scoring (corresponding to steps 2 and 3)

##### 3.1.2.1. General rules drawn up by the experts

The experts were aware that respondents may feel lethargic or “fatigued” when completing surveys. Hence, six approaches were applied to accommodate these potential effects:

1. The created items were presented to the respondent in four separate sections: BF, AP, EF, and one section for demographic information and PF. This was chosen so that the respondent quickly learns that he/she should focus on a single major concept (e.g., body functionality) for all of the items within the section.
2. In each section, the formulation of the items was harmonized meaning a unique question format was asked in each section followed by the desired concept of each item. For instance, all BF domain items were started with “*Do you have a problem with *…”.
3. Similarly, response options were unified for each section of the questionnaire. Case in point, the response options of all AP domain items were similar to the following, “*no difficulty/mild difficulty/moderate difficulty/severe difficulty/profound or complete difficulty”*.
4. If more than one item was linked to an ICF category, these questions were presented as consecutive items. For example, two items were linked to the category “Mental functions of language” (b167); H.16: “*Do you have a problem with understanding the meaning of a message in your language?”* and H.17: “*Do you have a problem with producing a meaningful message in your language?”*.
5. Where applicable, for the items in which their concept was very similar, but the aim of designing them was the distinction of the respondent's performance in different situations, the maximum effort was put to create a similar terminology for the common part of these items (e.g., the first part of three BF domain items (H.35, H.36, and H.37) which were all linked to the “Auditory perception” (b1560); “*Do you have a problem with understanding the speech of someone you know (your close family members and friends) *…*”).* The second part showed the difference between the three items; H.35 “… *over a distance of two or more meters?”*, H.36 “… *in a quiet environment?”*, H.37 “… *in a noisy environment?”*. This allowed a fair comparison to observe the effect of noise level and distance on speech perception, potentially excluding the other influential parameters and auditory cues in different scenarios.
6. As a result of cautiously choosing the terminology which reflects similar related categories, overall, 28 out of 90 (31%) of the ICF-based items were linked to more than one category (maximum up to five categories) which reduced the item redundancy to a high level.

Below, per section is described how the selected ICF categories were operationalized.

##### 3.1.2.2. Body functions

The items regarding body functionality started with “*Do you have a problem with *…*”.* This format was designed in such a way that it perfectly matched the recommended ICF qualifiers for impairments of the BF domain. Six approaches were applied to help the respondent better understand the context of the item and ease the choice of a response option. 1. The word “Problem” was used both in the item and response options. 2. The word “Impairment” was given in the response options along with the word “Problem” to further clarify the meaning. 3. The scale was also provided in numbers from zero to four. 4. Two options of “I don't know” and “Not applicable” were given to avoid any bias in the responses. 5. To avoid inducing the thoughts of having a problem, the passive term “*Do you have a problem with *…*”* was used rather than “*How much of a problem do you have with*…*”*. 6. The word “profound” was also added to the highest level of impairment (profound/complete problem/impairment). The complete problem is the ICF scaling system which reflects 95% of the problem/impairment. However, because “profound” is a more common term, it was added as an alternative.

As a result, the response options of the BF domain items were given as; “*0 (no problem/impairment)/1 (a mild problem/impairment)/2 (a moderate problem/impairment)/3 (a severe problem/impairment)/4 (a profound/complete* problem*/impairment)/ I don’t know/Not applicable”*. Among the eight chapters of the BF domain, the first three chapters were (partially) included as they were the most relevant ones to communication and conversation disability.

Chapter 1, Mental functions covered more global mental functions such as “Sleep functions” (b134) and specific mental functions such as “Mental functions of language” (b167) (items H.1, H.3, H.4–H.7, H.16, and H.17) and “Auditory perception” (b1560). Given that “Auditory perception” is highly relevant to the aim of this questionnaire, six items were developed to cover this category (items H.21–H.23 and H35–H.37).

Chapter 2, Sensory functions and pain: In total, 14 out of 48 BF domain items were developed to assess the “Hearing functions” (b230). All the third-level categories of b230 were covered separately, items H.24–H.35 and H.38–H.40. Besides, five items were developed to evaluate “Sensations associated with hearing and vestibular functions” (b240) (items H.12, H13, H.18–H.20).

Chapters 1 and 2, combined included 7 items addressing physical functioning/conditions other than those directly related to ear or hearing (items H.2, H.8–H.11, H.14, and H.15) along with one item from the AP domain (H.49). The items regarding hearing functioning and sound perception can be categorized into three groups based on the type of the sound of interest; non-speech sound (H.25–H.27, H.29, and H.31), exclusively speech sound (H.30, H.33, H.35–H.40), or general meaning the target sound could be speech or non-speech (H.21–H.24, H.28, H.32, and H.34).

Chapter 3, Voice and speech functions: Although none of the categories of this chapter is included in the brief ICF CSHL, all the categories of the comprehensive ICF CSHL of this chapter were included by creating eight items (H.41–H.48) These items were only needed for the respondents who reported having speech or voice impairments. This was inquired as H.41: “*Do you have any health conditions causing speech impairment or producing sounds? (e.g., caused by ENT problems, stroke, head injury, and other diseases)?”*. Since the respondent might not be able to rate this item precisely due to his/her disability, a specific formulation was applied to emphasize the fact that the rating is required based on the feedback/opinion of others and not the respondent's personal view. The items were formulated as “*If yes, have you been told by others that you have problems with *…*? How big was the problem from the other person's point of view?”*. As the category “Speaking” (d330) from the AP domain was found to be in line with the third chapter of the BF domain, the corresponding item was presented here (H.48).

Four categories that are not included in the BF domain in the two ICF CSHL but have been added to the questionnaire as recommended by the development team are: “Sleep functions” (b134), “Taste functions” (b250), and “Smell functions” (b255), “Lateralization of sound” (b2303) (6, 40, 47). For example, “Sleep functions” were found to be reported by individuals with HL and tinnitus. Further, since most family or social gatherings are dynamic, lateralization of sound is critical for successful communication and conversation activities. With regards to “Taste functions” and “Smell functions”, multiple sensory losses are common in individuals with HL. On one hand, HL is frequently described as a consequence or side effect of defined entities such as otological, cardiovascular, infectious, and neurological diseases. On the other hand, eating and drinking are common behaviors during family or social gatherings, which may influence how a person with HL functions when engaged in communication and conversation activities.

##### 3.1.2.3. Activities and participation

The design criteria of this domain were mainly similar to the BF. In the response options of the AP domain the term “*problem/impairment”* was replaced with “difficulty”. Nine items regarding different aspects of interaction, socialization, and relationships with different groups of people were developed (items H.50–H.55, H.58–H.60). Although these aspects might not be directly related to hearing functioning, they are essential to evaluate the disability degree of the individual's communication with the people whom he/she is in touch with the most.

The category “Undertaking multiple tasks” (d220) reflects the general concept of the ability to perform tasks. On the other hand, the following categories from the eighth chapter of the AP domain (Major life areas) relate to the ability to perform the assigned tasks in specific environments; “School education” (d820), “Higher education” (d830), “Remunerative employment” (d850), “Non-remunerative employment” (d855). To address these five categories and reduce the number of items, the following note was provided prior to the items to evaluate the difficulty with performing assigned tasks in general; “***Note:*** *Answer the questions H.63–H.66 with regards to the task you are assigned at your school, university, paid or unpaid work.”*.

Three categories that are not included in the AP domain of the comprehensive ICF CSHL were added: “Starting a conversation” (d3500), “Sustaining a conversation” (d3501), and “Ending a conversation” (d3502). As these categories directly address the conversation disability, they are included by combining their concept once with “Conversation with one person” (d3503) and once with “Conversation with many people” (d3504) (H.67 and H.68, respectively) to reduce the number of items.

##### 3.1.2.4. Environmental factors

The categories were chosen from all five chapters of EF. Three separate types of item formulation were considered to develop the items of this domain. The items referring to the factors that in nature are potentially a barrier to hearing and communication were constructed as *“What is the extent to which … can be considered a barrier?”.* As guidance, before presenting these items, the following general note was provided: “***Note:*** *When answering the questions H.81–H.86, think of a barrier as a hindrance, added difficulty, and restriction. Answer these questions considering the barrier that can affect your daily functioning/tasks (e.g., during listening-conversation activities)”*. For instance, the sound reverberation potentially is a barrier to performing communication tasks, therefore the question was formulated as H.85: “*What is the extent to which the reverberant or echoing environment (e.g., train station) can be considered a barrier?”.* The response options were provided as “*0 (no barrier)/1 (mild barrier)/2 (moderate barrier)/3 (severe barrier)/4 (profound/complete barrier)/I don’t know/Not applicable”*. In case, a respondent does not find a reverberant or echoing environment a barrier, still, three options are provided that can reflect his/her opinion against the initial assumption; “*0 (no barrier)/I don’t know/Not applicable”*.

The category “Light” (e240) was included (H.82) as patients with hearing disabilities can rely on visual cues such as lip-reading as a conversation technique. Therefore, the lack of light on the visual scene can be a barrier to daily functioning.

Where a factor is assumed to be a facilitator by its nature, the question was started with either “*What is the extent to which you rate the general support received from *…*?”* or “*What is the extent to which you rate the overall usefulness of *…*?”*. The word “support” was used where the item referred to people (e.g., family members or health professionals) given in H.75–H.79. The responses were provided as “*0 (no support)/1 (mild support)/2 (moderate support)/3 (substantial support)/4 (profound/complete support)/I don’t know/Not applicable”*. The word “usefulness” was used where the item referred to systems, services, policies, or hearing devices (H.80 and H.87–H.90). The response options were given as “*0 (no usefulness)/1 (mild usefulness)/2 (moderate usefulness)/3 (substantial usefulness)/4 (profound/complete usefulness)/I don’t know/Not applicable”*.

##### 3.1.2.5. Demographic information and personal factors

Thirty items were developed regarding demographic information, PF, and hearing status. The initial items inquire about the gender (A.1), age (A.2), marital status (A.3), current occupation status (A.4), years of education (A.5), attending school for hearing impaired students (A.6), and current living situation (A.7). Note that to inquire about the educational level (A.5), as it is expected that the questionnaire would be used in different countries with different educational systems (so as Germany and the USA), the educational level was asked with no offered response options and as a total number of years. The existence of a medical condition was asked to inquire about the general health status of the respondent (A.8). The following medical diagnoses were specifically asked and the respondent was required to add any other unspecified condition; Myocardial infarction, Peripheral vascular disease, Cerebrovascular accident (Stroke, Transient Ischemic Attack), Connective tissue disease, Diabetes mellitus, Hemiplegia, Chronic Kidney Disease/Dialysis, Liver disease, Cancer, and Chronic Obstructive Pulmonary Disease.

Medical interviews with 1,316 respondents performed at Hörzentrum Oldenburg gGmbH showed that the usage of firearms or exposure to loud noise for different purposes (either work-related or non-work-related) was the second most common self-reported possible causes of HL after age-related HL (40). Therefore, in this questionnaire, these incidences were inspected in detail (A.9–A.15).

Next, the HL status was inquired about here along with the main cause(s) of it (A.16 and A.17). The respondent was asked whether he/she experienced a sudden HL along with the age and cause of the incident (A.18). The history of ear surgery (A.19), the history of middle ear infection (A.20), an inquiry regarding suffering from running ear (A.21), and the last time that the respondent's hearing was evaluated (A.22) were requested next. Having a family member with HL (A.23), family side (A.24), and the exact relationship (A.25) were asked. The respondent then was asked if one of his/her ears hears better than the other one (A.26). The last four items were developed to clarify the hearing aid usage status. At first, the respondent was asked to mention if he/she uses a hearing aid (A.27), and if so, the type of the hearing aid (A.28), duration of hearing aid use (A.29), and the number of usage hours per day (A.30). The references used to design these 30 items are provided in Table 5.

**Table 5:** The sources used in developing the demographic information and Personal Factors items of the *HEAR-COMMAND Tool*.

Item number	Reference
A.1–A.8	(23, 24, 37)
A.9–A.15	(31, 39, 40)
A.16–A.17	(39)
A.18–A.20	(40)
A.21	(24, 31)
A.22	(31)
A.23–A.25	(24, 40)
A.26	−
A.27–A.30	(23, 24, 40)

The demographic information items are referred to as A.n where “n” represents the item number.

### 3.2. Content evaluation

#### 3.2.1. Questionnaire beta version; experts' perspectives (corresponding to step 4)

The beta version of the questionnaire in three languages (used in the data collection of this study) included 118 items of which 88 items were ICF-based and 30 items regarding demographic information.

#### 3.2.2. Questionnaire revised version; patients' perspectives (corresponding to step 5)

By applying the feedback received from the respondents and performing qualitative data analysis, the terminology of the items was revised accordingly in all languages where needed. Overall, the terminology of three demographic information and 27 ICF-based items was modified. The outcome of this approach (explained in the following section) yielded the creation of the revised version of the questionnaire. This version included 120 items of which 90 items were ICF-based and 30 items regarding demographic information and PF.

The complete revised *HEAR-COMMAND Tool* in English in a user-friendly format (as provided to the respondents in the USA) is provided in the Supplementary material (2).

##### 3.2.2.1. Respondents' characteristics

Table 6 illustrates the main respondents' characteristics across the three countries.

**Table 6:** The main demographic characteristics of the respondents in Germany, Egypt, and the USA.

Characteristics	Overall	Germany (German)	Egypt (Arabic)	USA (English)
Number of participants				
* N*	109	53	30	26
Gender				
Male	47	26	5	16
Female	62	27	25	10
Age				
Range	16–84	20–84	16–72	32–79
M ± SD	60.2 ± 17.8	68.9 ± 11.6	42.9 ± 18.1	62.6 ± 13.4
Education (years)				
M ± SD	14.6 ± 4.9	13.6 ± 4.4	13.3 ± 5.2	18.1 ± 4.1
Medical diagnosis				
Diagnosed/undiagnosed medical condition	62	34	11	17
No medical condition	45	17	19	9
Hearing status				
Aided				
Moderate to severe	28 (26%)	15	9	4
Mild	9 (8%)	5	0	4
Unaided				
Moderate to severe	25 (23%)	9	12	4
Mild	22 (20%)	10	5	7
Normal hearing	25 (23%)	14	4	7
Hearing aid type				
Receiver In Canal (RIC)	2	1	0	1
Invisible In Canal (IIC)	5	0	5	0
In The Ear (ITE)	2	2	0	0
Behind The Ear (BTE)	28	17	4	7
All other proposed types	0	0	0	0

##### 3.2.2.2. Qualitative analysis

The item-based feedback provided by the respondents yielded two types of item modifications; 1. An item was revised in all languages. 2. An item was unclear in one language and only reworded in that language and remained consistent as the beta version in other languages. The majority of the changes to the ICF-based items included adding more details, descriptions, or examples to the items for further clarification. This added terminology was either directly suggested by the respondents or decided by the experts based on the respondents' feedback. In the following, some of the modifications are provided. The most important changes commonly applied to all the languages of the beta version are provided in the Supplementary material (1), Table 3.

In some cases, the respondents claimed that it is difficult for them to distinguish if a functioning problem is directly caused by HL or if it can potentially have other causes. This confusion was reported mostly where impairment could be either caused by cognitive issues or HL. To clarify the matter, the following guidance was provided prior to presenting the ICF-based items in the revised version: “*The following questions relate to your general everyday life and can also relate to hearing, but do not have to. We have put a broader focus here, which can also go beyond hearing. When answering the questions, think about the last 30 days considering both healthy and worse days. If you use hearing technologies such as a hearing aid or cochlear implant or other hearing devices, please answer the way you hear with them.”*.

In the beta version, the items developed to evaluate the existence of a barrier were initially formulated as: “*Do you experience a barrier with …?”.* As the respondents found this terminology unclear or uncommon, it was changed to “*What is the extent to which … can be considered a barrier?”*.

The two additional ICF-based items in the revised version were the results of dividing two items of the beta version into separate items. In the beta version, items H.5 and H.6 were initially one single item as “*Do you have a problem with remembering things or recalling new information?”*. Items H.21 and H.22 were initially combined as “*Do you have a problem with distinguishing the pitch or tone of sounds?”.* In both cases, the respondents claimed that they have different impairment/problem degrees for the merged concepts and, consequently, the impairment degree should be reported separately.

According to the feedback provided by the respondents on the overall content of the tool, the items were found to be relevant to the targeted concepts (hearing, communication, and conversation disability).

## 4. Discussion

This study aimed to operationalize the ICF CSHL into a new self-rated questionnaire, named the *HEAR-COMMAND Tool*. The questionnaire was developed to evaluate hearing, communication, and conversation disability in adult patients with HL. The beta version of the questionnaire was designed and revised based on experts’ and patients' perspectives. The questionnaire includes 90 ICF-based items based on categories of the BF, AP, and EF domains. An additional set of 30 items were developed covering demographic information, hearing status, and PF. Except for BS, all the ICF categories of the brief ICF CSHL (a total of 85%) were covered. Moreover, the items covered 44% of the comprehensive ICF CSHL categories including 73% of the BF, 55% of AP, and 27% of EF domains. The results of the content evaluation in 109 patients present preliminary evidence to support the content validity of the questionnaire in adults seeking AR services and in different languages.

### 4.1. Methodological considerations

The ultimate aim is to improve the execution of audiological services, treatment, and rehabilitation for HL patients globally. That is to drive the individualized AR treatment plans that focus on maximizing residual capacity and performance of functioning, reducing activity limitations, and facilitating participation to the greatest extent possible. The goal of the functioning-based assessments is to evaluate the impairments and limitations experienced by individuals with HL in addition to the ear structural changes which can be captured by the audiological assessments and or medical examinations. The ICF-domain BS was therefore not included in the *HEAR-COMMAND Tool*. The results of the audiological evaluation coupled with the *HEAR-COMMAND Tool* can allow HHPs to determine if the objective HL is sufficient to create a functional communication deficit and if not to identify the potential factors requiring any additional investigations or referrals necessary. The results of the audiological assessments allow the audiologist to determine if the patient's HL is a result of the ear disease process and, if the severity of the HL creates an auditory functional deficit, and if so can the diminished auditory function explain the concerns of the individual being evaluated (e.g., problems in everyday functioning).

In many clinical cases, there is a mismatch between the audiological assessment and the self-evaluation results, hence, it is recommended that adults with HL should be evaluated *via* the biopsychosocial model (10,11, 19, 20). To evaluate the external and middle ear structure, audiological assessments including, but not limited to Otoscopy, Tympanometry/acoustic reflexes, and pure-tone audiometry can be considered. To evaluate inner ear structures and higher auditory functions, pure-tone audiometry, Otoacoustic emissions, speech, and word recognition testing, auditory brain stem response and cortical speech testing can be considered. Therefore, pairing the *HEAR-COMMAND Tool* with the audiological measurements and medical examination can yield a more ecological method to assess broad aspects of HL, communication, and conversation disability from a biopsychosocial framework (19–23, 48). The experts believe that the tool will help the HHPs to capture the needs of individuals with HL, establish the impact of HL on daily functioning, and monitor from which rehabilitative services the individual may derive benefits (49–51).

In 2019, Manchaiah et al., evaluated the content validity (e.g., the domains) of 14 patient-reported questionnaire instruments of hearing disability using the ICF as a classification reference (5). The results showed that the environmental and personal factors were not addressed as often as body functions, activities, and participation in the questionnaires. This may be explained by the fact that these earlier questionnaires were mostly designed according to the International Classification of Impairments, Disabilities and Handicaps (ICIDH) (52) where such psychosocial aspects are not magnified. The items of the *HEAR-COMMAND Tool* were developed according to the ICF framework which emphasizes the importance of the contextual factors in the success of the AR process and as a result, it can reveal a broader range of parameters correlating with the individual's disability and impairment.

The diversity of the instruments used in the development of the *HEAR-COMMAND Tool* can facilitate the comparability of available studies and the performance of meta-analyses (4, 5). The Alfakir questionnaire (23) and van Leeuwen e-Intake tool (24) were developed based on the brief ICF CSHL to screen or assess the functional status of patients with HL, which are necessary but not sufficient when it comes to the communication and conversation disability assessment. Many of the categories that are necessary to evaluate the communication ability by targeting the difficulties in conducting relationships or conversation disability are not included in the brief ICF CSHL but are listed in the comprehensive ICF CSHL, which are included in the *HEAR-COMMAND Tool*.

The questionnaire was evaluated in three countries and in three different languages to accommodate a wide range of respondents; however, the target population did not necessarily reflect a representative sample. For instance, in the subgroup of aided respondents, the used hearing aid types were either “Receiver In Canal (RIC)”, “Invisible In Canal (IIC)”, “In The Ear (ITE)”, or “Behind The Ear (BTE)”. The target population did not include the respondents using other types of hearing devices including “Cochlear Implant (CI)” and “Bone-Anchored Hearing Aid (BAHA)”. Furthermore, the population did not include any respondents with HL caused by “accidents or skull injury” or “Infectious disease”. For each of the following HL causes, only one respondent was included: “Congenital”, “Ototoxicity”, and “ear surgery”.

### 4.2. Clinical and research implications

The ICF is a part of the WHO family of international classifications network (53, 54) including the International Classification of Diseases (ICD) (55) and the International Classification of Health Interventions (ICHI) (56). Currently, there is an ongoing effort to link the ICF with the ICD-11 (57) and ICHI entities (58, 59). This allows the professional to describe the additional details about the impairments, disabilities, and interventions (medical, surgical, mental health, primary care, allied health, functioning support, rehabilitation, traditional medicine, and public health) relevant to HL. The *HEAR-COMMAND Tool* has the potential to be applied as a midpoint approach between the WHO's classification network. For example, in the ICF, functioning represents not only an outcome but also the starting point of the clinical assessment, intervention management, post-intervention evaluation, and quality management. Importantly, collecting information on the functioning and addressing functioning information relevant to rehabilitation needs by utilizing the ICF is one of the 10 priority identified areas for action reported by the “Rehabilitation 2030 initiative call of action” (60). The other identified areas include, (1) developing a strong, multidisciplinary rehabilitation workforce that is suitable for each country's context and ensuring rehabilitation as a topic is included in all health workforce education efforts, (2) establishing and strengthening networks and partnerships in rehabilitation, particularly between low-middle- and high-income countries like Egypt, and (3) building research capacity and expanding the availability of quality evidence for rehabilitation. The Rehabilitation 2030 initiative draws attention to the profound unmet need for rehabilitation worldwide and highlights the importance of strengthening health systems to provide a global rehabilitation community. As such, the experts believe that the development of the *HEAR-COMMAND Tool* is aligned with the Rehabilitation 2030 initiative areas.

The *HEAR-COMMAND Tool* has the potential to complement previous questionnaire tools on outcome quality in auditory rehabilitation, such as the SSQ (34), but also hearing-specific and generic health-related quality of life inventories [e.g., see (61)] in the field of cochlear implantation or hearing aid evaluation (62). This tool can be considered the core for evaluating the hearing, communication, and conversation disability among people living with HL, yet, it has the potential for “add-on options”. For example, some ICF categories have been identified by the previous studies using the ICF as a reference system; yet they were not considered in the questionnaire design—such as “Intellectual functions”, “Energy level”, “Motivation”, “Visual perception”, and “Motor-related functions and activities” (6, 46). Hence, there is an ongoing study to determine the effectiveness of pairing the tool with the audiological measurements to determine the applicability of incorporating the above categories into the tool.

The overall aim of this project is to improve the execution of audiological services, treatment, and rehabilitation for adult patients with HL. As a first step, a new questionnaire was developed grounded on the broad perspective of the ICF CSHL to assess the hearing, communication, and conversation disability in individual adults with HL. The development was based on multinational and professional experts' and patients' perspectives. The pilot validation study in patients showed positive results with regard to the HEAL-COMMAND tool's content validity, including its relevance to identifying communication-related problems. The findings are promising for the further optimization of a tool that has the potential to facilitate improvement in the execution of audiological services, treatment, and rehabilitation for HL patients.

## Data Availability

The datasets presented in this study can be found in online repositories. The names of the repository/repositories and accession number(s) can be found below: https://zenodo.org/record/6906963#.YuAmAYRByUk. Title: “The visualization of the collected data corresponding to the following paper: “The development of a Self-Rated ICF-based questionnaire (HEAR-COMMAND Tool) to evaluate Hearing, Communication, and Conversation disability: multinational experts’ and patients’ perspectives”” (63). Zenodo website. Version 1. DOI: 10.5281/zenodo.6906962.
