# Supplementary material for: The development of a Self-Rated ICF-based questionnaire (HEAR-COMMAND Tool) to evaluate Hearing, Communication, and Conversation disability: Multinational experts’ and patients’ perspectives
Source: Front Rehabil Sci. 2022 Nov 14;3:1005525. doi: 10.3389/fresc.2022.1005525 (PMC9701723; doi:10.3389/fresc.2022.1005525)
Supplement: Supplementary file 1 [file Datasheet1.pdf]

Table 1: The ICF-based developed items of the *HEAR-COMMAND Tool* and the corresponding linked ICF category along with the used resources.

Symbol guidance: ⊕ (Modified item) To develop this item, new words/terms were added to the existing source(s). Ө (New item) no reference was used and the question is newly developed. O (Original item) No major change was applied to the existing source(s).

Body Functions		
Do you have a problem with ...		
b126	Temperament and personality functions	-
Ө **H.1** changing your attitude or mood when you are in an unpredictable or stressful situation? (Also linked to d240)		
b134	Sleep functions	WHS , Van Leeuwen Tool
O **H.2** sleeping (falling asleep, waking up often during the night, or waking up early in the morning)?		
b140	Attention functions	WHODAS 2.0, SSQ, DHI, Alfakir questionnaire, OQUA
⊕ **H.3** focusing your attention on one thing? (Also linked to d160)<br>O **H.4** maintaining your focus on two or more things at the same time? (Also linked to d160)		
b144	Memory functions	WHS, Alfakir questionnaire
O **H.5** remembering things?<br>O **H.6** recalling new information?		
b152	Emotional functions	WHS, DHI, Alfakir questionnaire, OQUA
O **H.7** sadness or depression?		
b1560	Auditory perception	Alfakir questionnaire
Ө **H.21** distinguishing the pitch of sounds?<br>Ө **H.22** distinguishing the tone of sounds?<br>Ө **H.23** distinguishing the volume of sounds? (Also linked to e2500)<br>⊕ **H.35** understanding the speech of someone you know (your close family members and friends) over distance of two or more meters?<br>Ө **H.36** understanding the speech of someone you know (your close family members and friend) in a quiet environment?<br>⊕ **H.37** understanding the speech of someone you know (your close family members and friend) in a noisy environment? (Also linked to b140/d160/e2501)		
b167	Mental functions of language	-
Ө **H.16** understanding the meaning of a message in your language?<br>Ө **H.17** producing a meaningful message in your language?		
b210	Seeing functions	WHODAS 2.0
O **H.8** seeing and recognizing a person you know across the road (with glasses or contact lenses, if necessary)?<br>O **H.9** seeing and recognizing an object at arm's length (with glasses or contact lenses, if necessary)?		
b2300	Sound detection	AIAHD
Ө **H.24** detecting a sound in your surrounding environment?<br>O **H.25** detecting noises in the household, like running water or a washing machine?		
b2301	Sound discrimination	SSQ, AIAHD
O **H.26** discriminating the sound of a car and a bus? (Also linked to b1560)		

<table>
<tr><td colspan="3">O H.27 recognizing which instruments are playing when you are listening to music? (Also linked to b1560)
O H.34 hearing a single jumbled sound when you are hearing more than one sound at the time? (Also linked to b1560)</td></tr>
<tr><td>b2302</td><td>Localisation of sound source</td><td>SSQ, AIAHD</td></tr>
<tr><td colspan="3">Θ H.28 detecting where a sound comes from?
O H.29 telling whether a bus or truck is coming towards you or going away?
O H.30 detecting from what corner of a lecture room someone is asking a question during a meeting?
O H.31 telling how far away a bus or a truck is from the sound?
⊕ H.32 telling where a human is when he screams or where a dog is when it barks loudly without having to look?</td></tr>
<tr><td>b2303</td><td>Lateralization of sound</td><td>SSQ</td></tr>
<tr><td colspan="3">O H.33 detecting right away whether the person on your left or the person on your right starts talking, without having to look?</td></tr>
<tr><td>b2304</td><td>Speech discrimination</td><td>SSQ, AIAHD</td></tr>
<tr><td colspan="3">O H.38 understanding the presenter of the news on the radio or TV? (Also linked to b140/b1560/d115/d160/d310)
O H.39 understanding what someone is saying while the TV is on at the same time without turning the TV down? (Also linked to b140/b1560/d115/d160/e2501)
⊕ H.40 understanding the presenter of the news on the radio or TV and understanding what someone is saying at the same time? (Also linked to b140/b1560/d115/d160/e2501)</td></tr>
<tr><td>b240</td><td>Sensations associated with hearing and vestibular functions</td><td>NHANES, Alfakir questionnaire, Van Leeuwen Tool, OQUA</td></tr>
<tr><td colspan="3">⊕ H.12 dizziness when standing or changing positions or walking or even when your head is still?
⊕ H.13 your balance when standing or walking or changing position (e.g., being unsteady, off-balance)?
O H.18 ringing, beeping, roaring, or buzzing in your ears?
⊕ H.19 a feeling of pressure or pressure balance in your ear (“popping” of the ear) in your daily life?
O H.20 irritation (e.g., itching) on or in your ear?</td></tr>
<tr><td>b250</td><td>Taste function</td><td>Van Leeuwen Tool, OQUA</td></tr>
<tr><td colspan="3">O H.10 taste loss?</td></tr>
<tr><td>b255</td><td>Smell function</td><td>Van Leeuwen Tool, OQUA</td></tr>
<tr><td colspan="3">O H.11 smell loss?</td></tr>
<tr><td>b280</td><td>Sensation of pain</td><td>WHS</td></tr>
<tr><td colspan="3">⊕ H.14 pain in general?
Θ H.15 pain in your head and neck area? If you have problems, please specify the exact area in which you feel the pain. Which area?...</td></tr>
<tr><td>b310</td><td>Voice functions</td><td>-</td></tr>
<tr><td colspan="3">Θ H.41 Do you have any health conditions causing speech impairment or producing sounds? (e.g., caused by ENT problems, stroke, head injury and other diseases)? If no, continue with question H49!
Θ H.42 If yes, have you been told by other people that you have problems with whistling or making other sounds (not speech) with your mouth? How big was the problem from the other persons’ point of view?
Θ H.43 If yes, have you been told by others that you have problems with changing the pitch of sounds (other than speech), e.g., whistles? How big was the problem from the other persons’ point of view?
Θ H.44 If yes, have you been told by others that you have problems with changing the volume of sounds (other than speech) that they make, such as whistling? How big was the problem from the other persons’ point of view?</td></tr>
<tr><td>b320</td><td>Articulation functions</td><td>-</td></tr>
<tr><td colspan="3">Θ H.45 If yes, have you been told by other people that you have problems with pronunciation? How big was the problem from the other persons’ point of view?
Θ H.46 If yes, have you been told by other people that you have problems with regulating the volume of your speech (too soft or too loud)? How big was the problem from the other persons’ point of view?</td></tr>
<tr><td>b330</td><td>Fluency and rhythm of speech functions</td><td>-</td></tr>
<tr><td colspan="3">Θ H.47 If yes, have you been told by other people that you have problems with the speed of your speech? How big was the problem from the other persons’ point of view?</td></tr>
<tr><td>d330*</td><td>Speaking</td><td>-</td></tr>
<tr><td colspan="3">Θ H.48 If yes, have you been told by other people that you have problems with telling stories or reporting on something? How big was the problem from the other persons’ point of view? (*This question is linked to d330, but based on the concept, it’s inserted here)</td></tr>
<tr><td colspan="3">Activities and Participation</td></tr>
</table>

Do you have difficulty with ...		
d110	Watching	-
**H.90**		
d115	Listening	Alfakir Questionnaire
O **H.74** listening to the television, radio, or music? (Also linked to b1560)		
d160	Focusing attention	-
**H.3**, **H.4**		
d220	Undertaking multiple tasks	-
**H.63**, **H.64**, **H.65**, **H.66**		
d240	Handling stress and other psychological demands	WHODAS 2.0, Van Leeuwen Tool
O **H.49** dealing with stressful situations? (Also linked to b126)		
d310	Communicating with - receiving - spoken messages	Alfakir Questionnaire
⊕ **H.57** understanding a statement or question during communication activity?		
d330	Speaking	-
**H.48**		
d3500<br>d3501<br>d3502	Starting a conversation<br>Sustaining a conversation<br>Ending a conversation	-
Merged with **H.67** and **H.68**		
d3503	Conversing with one person	SSQ, SAC, AIAHD
Θ **H.67** starting, continuing, or ending a conversation, or speaking with someone? (Merged with d3500, d3501, d3502)<br>O **H.69** carrying on a conversation with someone during a crowded meeting? (Also linked to b140/d160/e2501)<br>O **H.70** carrying on a conversation with somebody in a bus or car? (Also linked to b140/d160/e2501)<br>O **H.73** telling what someone is saying when the conversation switches from one person to another? (Also linked to b140/d160/e2501)		
d3504	Conversing with many people	WHODAS 2.0, Alfakir Questionnaire, SAC, AIAHD
O **H.68** starting, continuing, or ending a conversation, or speaking with several people in a group? (Merged with d3500, d3501,d3502)<br>O **H.71** following a conversation between five people in a busy restaurant while you can see everyone? (Also linked to b140/d160/e2501)		
d355	Discussion	-
Θ **H.56** starting, continuing, or ending an argument or debate with one person or many people?		
d360	Using communication devices and techniques	AIAHD
Θ **H.62** performing communication techniques such as lip-reading? (Also linked to e240)<br>O **H.72** carrying a phone call in a quiet room?		
d710	Basic interpersonal interactions	-
Θ **H.50** interacting with people in a socially appropriate manner (e.g. regulating emotions, controlling verbal and physical aggression)?		
d720	Complex interpersonal interactions	-
Θ **H.61** starting and continuing relationships in a socially appropriate manner (e.g. regulating emotions, controlling verbal and physical aggression)?		
d730	Relating with strangers	WHODAS 2.0
O **H.52** dealing with people you do not know?		

d740	Formal relationships	-
Θ **H.53** starting and continuing formal relationships with people in authority (e.g. employers, professionals or service providers)?		
d750	Informal social relationships	WHODAS 2.0, Alfakir Questionnaire
Θ **H.51** socializing with people living in your community (e.g. Classmates, co-workers)?<br>O **H.54** socializing with your family or friends?<br>O **H.55** making new friends? (Also linked to d720)		
d760	Family relationships	Alfakir Questionnaire, Van Leeuwen Tool
O **H.58** maintaining relationships with your immediate family members (parents, partner, children)?		
d820<br>d830<br>d850<br>d855	School education<br>Higher education<br>Remunerative employment<br>Non-remunerative employment	WHODAS 2.0
**Note:** Answer the questions H.63 – H.66 with regards to the task you are assigned at your school, university, paid or unpaid workplace.<br>O **H.63** your day-to-day occupation/tasks? (Also linked to d220)<br>O **H.64** doing your most important tasks well? (Also linked to d220)<br>O **H.65** getting done all the tasks that you needed to do? (Also linked to d220)<br>O **H.66** getting your tasks done as quickly as needed? (Also linked to d220)		
d910	Community life	WHODAS 2.0
O **H.59** joining in community activities (for example, festivities, religious or other activities) in the same way as anyone else can?		
d920	Recreation and leisure	-
Θ **H.60** engaging in any hobby or pleasurable activity (such as games, sports, or going to cinema)?		

Environmental Factors

I: Usefulness: What is the extent to which you rate the overall usefulness of …?
II: Support: What is the extent to which you rate the general support received from …?
III: Barrier: What is the extent to which the … can be considered a barrier?

e125	Products and technology for communication	Alfakir questionnaire, Van Leeuwen Tool
**Please answer questions H.87 to H.90 only if you have hearing aids!**<br>O **H.87** (I) your hearing aid in your normal daily routines?<br>O **H.88** (I) your hearing aid during listening-conversation activities?<br>Θ **H.89** (I) your hearing aid while using a telephone or cellphone?<br>Θ **H.90** (I) your hearing aid while watching TV? (Also linked to d110)		
e150	Design construction and building products and technology of buildings for public use	-
Θ **H.81** (III) design and construction of your workplace/task place		
e240	Light	-
Θ **H.82** (III) darkness or the absence of light … (e.g. in lipreading)		
e2500	Sound intensity	-
Θ **H.83** (III) low volume of speech (Also linked to b1560)		
e2501	Sound quality	SAC, Alfakir questionnaire

O **H.84** (III) background noise (Also linked to b1560/b2304)<br>Θ **H.85** (III) reverberant or echoing environment (e.g., train station) (Also linked to b1560/b2304)<br>Θ **H.86** (III) unclear sound (Also linked to b1560)		
e310<br>e320	Immediate family<br>Friends	Alfakir questionnaire, Van Leeuwen Tool
O **H.77** (II) your close family members and friends in your daily functioning especially during listening-conversation activities?		
e355	Health professionals	Van Leeuwen Tool
O **H.79** (II) your most important healthcare professional(s)?		
e410<br>e420	Individual attitudes of immediate family members<br>Individual attitude of friends	-
Θ **H.76** (II) your close family members and friends (such as providing emotional and social support, encouragement and so on)?		
e460	Societal attitudes	SAC, Van Leeuwen Tool
⊕ **H.75** (II) people in your society (such as providing emotional and social support, encouragement etc.)?		
e535	Communication services, systems and policies	-
Θ **H.80** (I) the communication services and systems you use daily such as telephone, cellphone, speaker, bluetooth connection and so on?		
e580	Health services, systems and policies	-
Θ **H.78** (II) the main health services and systems offered in relation to your hearing aids and medical services (e.g. ear specialist)?		

Table 2: The references used in table 1 illustrating the 13 resources used in the design of the *HEAR-COMMAND Tool*

**Questionnaire**	**Abbreviation**	**Developer(s)**	**Reference**
National Health And Nutrition Examination Survey-Audiometry	NHANES	United States Centers for Disease Control and Prevention (CDC) 2007-2008	31
World Health Organization (WHO) Disability Assessment Schedule	WHODAS 2.0	Üstün et al. and WHO 2010	32
World Health Survey Individual Questionnaire	WHS	WHO 2002	33
The Speech, Spatial and Qualities of hearing scale	SSQ	Gatehouse & Noble 2004	34
Dizziness Handicap Inventory	DHI	Jacobson et al. 1990	35
Self-Assessment of Communication	SAC	Schow & Nerbonne 1982	36
Self-Assessments ICF Core Sets for Hearing Loss Questionnaire	Alfakir Questionnaire	Alfakir & Holmes 2017	23
An ICF-Based e-Intake Tool in Clinical Otology and Audiology Practice	van Leeuwen e-Intake Tool	van Leeuwen et al. 2020a	24
The ICF checklist Version 2.1a	ICF checklist	WHO 2007	37
Otology Questionnaire Amsterdam	OQUA	Bruinewoud et al. 2018	38
Hearing, Lifestyle and Health Questionnaire (Fragebogen Hören, Lebensgewohnheiten und Gesundheit)	HLHQ	Hörzentrum Oldenburg gGmbH, Gieseler et al., 2017	39
Ohrstrom HL medical interview	OHL	Hörzentrum Oldenburg gGmbH, gathered in Afghah, et al. 2022	40
Amsterdam Inventory for Auditory Disability and Handicap	AIADH	Kramer et al. 1995	41

[23] Alfakir, R., & Holmes, A. E. (2017). Development and Validation of a Questionnaire Measuring Functioning Abilities of Older Adults Living with Hearing Disability: Implications for Audiologic Rehabilitation. *Journal of the Academy of Rehabilitative Audiology*, *50*.

[24] van Leeuwen, L. M., Pronk, M., Merkus, P., Goverts, S. T., Terwee, C. B., & Kramer, S. E. (2020a). Operationalization of the Brief ICF core set for hearing loss: An ICF-Based e-intake tool in clinical otology and audiology practice. *Ear and hearing*, *41*(6), 1533. DOI: 10.1097/AUD.0000000000000867.

[31] Centers for Disease Control and Prevention (CDC). National Center for Health Statistics (NCHS). National Health And Nutrition Examination Survey questionnaire (NHANES), Hyattsville, MD, U.S., Department of Health and Human Services, Centers for Disease Control and Prevention, 2007-2008. https://www.cdc.gov/nchs/nhanes/index.htm. (Accessed: May 21, 2022).

[32] Üstün, T. B., Kostanjsek, N., Chatterji, S., & Rehm, J. (Eds.). (2010). *Measuring health and disability: Manual for WHO disability assessment schedule WHODAS 2.0*. World Health Organization.

[33] World Health Organization (WHO). World Health Survey (WHS) 2002; Individual questionnaire. https://apps.who.int/healthinfo/systems/surveydata/index.php/catalog/whs/about. (Accessed: May 21, 2022).

[34] Gatehouse, S., & Noble, W. (2004). The speech, spatial and qualities of hearing scale (SSQ). *International journal of audiology*, *43*(2), 85-99. https://doi.org/10.1080/14992020400050014.

[35] Jacobson, G. P., & Newman, C. W. (1990). The development of the dizziness handicap inventory. *Archives of Otolaryngology–Head & Neck Surgery*, *116*(4), 424-427. DOI: 10.1001/archotol.1990.01870040046011.

[36] Schow, R. L., & Nerbonne, M. A. (1982). Communication screening profile: Use with elderly clients. *Ear and Hearing*, *3*(3), 135-147.

[37] World Health Organization (WHO). (2007). ICF Checklist version 2.1 a, clinician form. *https://www.who.int/publications/m/item/icf-checklist*. (Accessed: May 21, 2022).

[38] Bruinewoud, E. M., Kraak, J. T., van Leeuwen, L. M., Kramer, S. E., & Merkus, P. (2018). The Otology Questionnaire Amsterdam: a generic patient reported outcome measure about the severity and impact of ear complaints. A cross-sectional study on the development of this questionnaire. *Clinical Otolaryngology*, *43*(1), 240-248. DOI: 10.1111/coa.12950

[39] Gieseler, A., Tahden, M. A., Thiel, C. M., Wagener, K. C., Meis, M., & Colonius, H. (2017). Auditory and non-auditory contributions for unaided speech recognition in noise as a function of hearing aid use. Frontiers in psychology, 8, 219. https://doi.org/10.3389/fpsyg.2017.00219.

[40] Afghah, T., Schütze, J., Meis, M., Kollmeier, B., & Wagener, K. C. (2022). Conformities and gaps of clinical audiological data with the international classification of functioning disability and health core sets for hearing loss. *International Journal of Audiology*, 1-10. https://doi.org/10.1080/14992027.2022.2078433.

[41] Kramer, S. E., Kapteyn, T. S., Festen, J. M., & Tobi, H. (1995). Factors in subjective hearing disability. *Audiology*, *34*(6), 311-320.

Table 3: The modifications applied to the beta version after data collection to create the revised version of the *HEAR-COMMAND Tool*

Nr.	Beta version	Revised version	Note
**A.14**	Examples include noise from power tools, lawn mowers, farm machinery, cars, trucks, motorcycles, motor boats, or loud music	For example engine noise or loud music	The participants claimed that the question is clear and that providing 8 examples is unnecessary.
**A.17**	If yes, select the main causes:<br>2 Noise-induced hearing loss<br>3 Music event	2 Noise-induced hearing loss (excluding music events or concerts)<br>3 Noise-induced hearing loss caused after music events or concerts	The participants claimed that the response option "Noise-induced hearing loss" can potentially include a music event, therefore there is an overlap between the two response options.
**A.28**	1 Behind The Ear (BTE)<br>2 In-The-Ear (ITE)<br>3 In-The-Canal (ITC)<br>4 Invisible In Canal (IIC)<br>5 Completely In Canal (CIC)<br>6 Mini Canal (MC)<br>7 Receiver In Canal (RIC)<br>8 Microtube<br>9 Cochlear Implant (CI)<br>10 Contralateral Routing Of Signals (CROS)<br>11 Bilateral microphones with Contralateral Routing Of Signal (BiCROS)<br>12 Bone-Anchored Hearing Aid (BAHA)<br>13 Others (Please specify)	1 Hearing aid (including Behind-The Ear, In-The-Ear, Receiver In Canal, Bone- Anchored Hearing Aid; BAHA)<br>2 Cochlear Implant (CI)<br>3 Others (Please specify)	The collected data showed that many of the options were not chosen in any country, so the response options were limited to hearing aid.
**H.01**	Do you have a problem with adjusting (correcting or modifying or compromising) your attitude or mood during a stressful or unexpected situation?	Do you have a problem with changing your attitude or mood when you are in an unpredictable or stressful situation?	The participants claimed that the words "correcting or modifying or compromising" were found to be unclear and lengthy.
**H.05 and H.06**	Do you have a problem with remembering things or recalling new information?	Do you have a problem with remembering things?<br>Do you have a problem with recalling new information?	The participants claimed that they have a different degree of impairment/problem for the combined concepts.
**H.12 and H.13**	Do you have a problem with dizziness?<br>Do you have a problem with loss of balance?	Do you have a problem with dizziness when standing or changing positions or walking or even when your head is still?<br>Do you have a problem with your balance when standing or walking or changing position (e.g., being unsteady, off-balance)?	The participants claimed that the differentiation of the terms "dizziness" and "loss of balance" is difficult and unclear. More explanation is needed.

**H.16 and H.17**	Do you have a problem with understanding a spoken or written message in your language?<br>Do you have a problem with producing a meaningful spoken or written message in your language?	Do you have a problem with understanding the meaning of a message in your language?<br>Do you have a problem with producing a meaningful message in your language?	The questions were asked to target the cognitive aspect of understanding a language. The questions were linked to “Mental functions of language” (b167). Using the words “spoken or written” misled the participants.
**H.19**	a feeling of pressure or pressure balance in your ear (“popping” of the ear)?	…(“popping” of the ear) in your daily life?"	The participants claimed that they were not certain about the targeted time period.
**H.21 and H.22**	Do you have a problem with distinguishing the tone or pitch of sounds?	Do you have a problem with distinguishing the pitch of sounds?<br>Do you have a problem with distinguishing the tone of sounds?	The participants claimed that they have a different degree of impairment/problem for the combined concepts.
**H.32**	Do you have a problem with telling where a dog is when it barks loudly from its sound and without having to look?	Do you have a problem with telling where a human is when he screams or where a dog is when it barks loudly without having to look?	The participants recommended using an additional example to clarify the target meaning.
**H.35, H.36 and H.37**	Do you have a problem with understanding the speech of someone you know (i.e., spouse, partner, child, sibling, or close friend, health care provider, etc.) over a distance/in a quiet environment/ in a noisy environment?	Do you have a problem with understanding the speech of someone you know (your close family members and friends) over distance of two or more meters?	The participants claimed that the aimed “Distance” was not clear.<br>The examples were not necessary.
**Prior to H.63**	Note: When answering the following four questions, do not think of private activities, but of more public tasks, such as education, work or volunteering.	Note: Answer the questions 63 - 66 with regards to the task you are assigned at your school, university, paid or unpaid workplace.	The participants claimed that the guidance sentence needed more clarification.
**H.75 and H.76**	What is the extent to which you rate the support received from people in your society/family members (attitude, opinion, and beliefs)?	What is the extent to which you rate the support received from people in your society/family members (such as providing emotional and social support, encouragement etc.)?	The participants claimed that the terms “attitude, opinion, and beliefs” were found to be too general, and instead providing examples is more helpful.
**H.76 and H.77**	close family members	close family members and friends	The participants who do not live with family members claimed that the question is meaningful if it’s asked about a close friend.
**H.80**	What is the extent to which you rate the usefulness of the communication services and systems you use daily such as telephone and cellphone?	What is the extent to which you rate the usefulness of the communication services and systems you use daily such as telephone, cellphone, speaker, Bluetooth connection and so on?	The participants claimed that more examples of the communication services and systems were needed.
**H.81 to H.86**	Do you experience a barrier with …?	Note: When answering the questions 81 - 86, think of a barrier as a hindrance, added difficulty, and restriction. Answer these questions considering the barrier that can affect your daily functioning/tasks (e.g., during listening-conversation activities).	As the participants found this terminology unclear or uncommon, the item formulation was changed in all of the questions with barrier terms.

		What is the extent to which … can be considered a barrier?	
**H.82**	Do you experience a barrier with the absence of light in daily functioning (e.g. in lip-reading)?	What is the extent to which the darkness or the absence of light can be considered a barrier (e.g., in lip-reading)?	Adding the word “darkness” helps better understand the term “the absence of light”.
**H.85**	Do you experience a barrier with the reverberant (echoing) environment during listening-conversation activities?	What is the extent to which the reverberant or echoing environment (e.g., train station) can be considered a barrier?	The participants claimed that adding an example of an echoing environment would further clarify the question.
**H.86**	Do you experience a barrier or restriction when everyday sounds are not clear?	What is the extent to which the unclear sound can be considered a barrier?	The participants claimed that the term “everyday sound” was not clear.
