# Supplementary material for: The development of a Self-Rated ICF-based questionnaire (HEAR-COMMAND Tool) to evaluate Hearing, Communication, and Conversation disability: Multinational experts’ and patients’ perspectives
Source: Front Rehabil Sci. 2022 Nov 14;3:1005525. doi: 10.3389/fresc.2022.1005525 (PMC9701723; doi:10.3389/fresc.2022.1005525)
Supplement: Supplementary file 2 [file Datasheet2.pdf]

Personal Information

A01	Please enter your gender.		
	Female	Male	Diverse
	1 ☐	2 ☐	3 ☐

A02	What's your date of birth?
	______________________(Month/Day/Year)

A03	What is your current marital status?					
	Never married	Currently married	Divorced	Widowed	Separated	Cohabiting
	1 ☐	2 ☐	3 ☐	4 ☐	5 ☐	6 ☐

..

A04	What is your current occupation?
	1 ☐ Paid employment<br>2 ☐ Self-employed<br>3 ☐ Student<br>4 ☐ Non-paid work (such as volunteer, charity)<br>5 ☐ Keeping house/House-maker<br>6 ☐ Retired<br>7 ☐ Unemployed (health reason) **Please specify:** ______________________<br>8 ☐ Unemployed (other reason) **Please specify:** ______________________<br>9 ☐ Others; **Please specify:** ______________________

A05	**How many years were you educated in school and university/professional training combined?**
	My education included a total of ___________ years.

A06	**Have you attended a school for the deaf/hearing impaired?**	
	Yes	No
	1 ☐	2 ☐

A07	**What is your current living situation?**
	1 ☐ I live with my partner.<br>2 ☐ I live without a partner but with my child/children.<br>3 ☐ I live together with my partner and child/children.<br>4 ☐ I live with one or more friends.<br>5 ☐ I live with one or more family members (e.g., parents, siblings).<br>6 ☐ I live with other unrelated individuals (e.g., roommate)<br>7 ☐ I live alone, independently.<br>8 ☐ Others; **please specify:** _____________________

A08	**Please state all medical diagnoses regarding your current state of health. The following medical condition pre-exists in the last 12 months or lasts up to 12 months after diagnosis: (If possible, Select the given example)**

1 ❑ No medical condition

2 ❑ The following medical condition:

1 ❑ **Mental and cognitive disorders**
(e.g., Anxiety, Depression, Bipolar Personality, Mild Cognitive Impairment, Dementia)

2 ❑ **Sensory disorders and pain**
(e.g., Visual loss, Vestibular and Balance loss, Neuropathy)

3 ❑ **Voice and speech disorders**
(e.g., Hoarseness, Vocal fold paralysis)

4 ❑ **Cardiovascular disease**
(e.g., Myocardial infarction, Peripheral vascular disease, Heart failure, Heart attack, Elevated blood pressure)

5 ❑ **Neurological disease**
(e.g., Stroke, Transient Ischemic Attack, brain tumors)

6 ❑ **Hematological disease**
(e.g., lymphocytic leukemia, Amyloidosis, Anemia)

7 ❑ **Immunological disease**
(e.g., Rheumatoid arthritis, Inflammatory bowel disease, Multiple Sclerosis)

8 ❑ **Respiratory disease**
(e.g., Asthma, chronic obstructive pulmonary disease (COPD), Long-term COVID-19, Lung cancer)

9 ❑ **Digestive disease**
(e.g., Gastroesophageal reflux disease, Ulcers)

10 ❑ **Metabolic disease**
(e.g., Elevated fat/cholesterol levels, Obesity, Elevated blood sugar, Insulin resistance, Diabetes mellitus)

11 ❑ **Endocrine disease**
(e.g., Hyperthyroidism, Hypothyroidism, Chronic Kidney Disease / Dialysis, Liver disease)

12 ❑ **Neuromusculoskeletal and movement disease**
(e.g., Parkinson's disease, Falling)

	13 ❑ **Others; please specify:**<br>______________________________<br>______________________________<br>______________________________<br>______________________________<br><br>**What prescribed medications do you take every day?**<br>______________________________<br>______________________________<br>______________________________

**A09**	**Have you ever used firearms for target shooting or hunting for work or military service?**		
	Yes, Several times	Yes, Sometimes	No
	1 ☐	2 ☐	3 ☐

→ If No, go to question A11

**A10**	**If yes, how often did you wear hearing protection (earplugs, earmuffs) when shooting with firearms?**				
	Never (or almost never)	Rarely	About half the time	Usually	Always (or almost always)
	0 ☐	1 ☐	2 ☐	3 ☐	4 ☐

**A11**	**Have you ever had a job where you were exposed to noise or loud noise, for five or more hours a week (e.g. engine noise or loud music)?**			
	Yes	No	I don't know	Not applicable (never worked)
	1 ☐	2 ☐	3 ☐	4 ☐

→ If No, go to question A14

A12	If yes, how many months or years have you been exposed to?							
	Less than 3 Months	3 – 11 Months	1 – 2 Years	3 – 4 Years	5 – 9 Years	10 – 14 Years	15 Or more Years	I don’t know
	1 ☐	2 ☐	3 ☐	4 ☐	5 ☐	6 ☐	7 ☐	8 ☐

A13	If yes, how often have you worn hearing protection (earplugs, earmuffs)?				
	Never (or almost never)	Rarely	About half the time	Usually	Always (or almost always)
	0 ☐	1 ☐	2 ☐	3 ☐	4 ☐

A14	Outside of your job, were you ever exposed to noise or loud noise, for five or more hours a week (e.g. engine noise or loud music)?		
	Yes	No	I don’t know
	1 ☐	2 ☐	3 ☐

→ **If No, go to question A16**

A15	If yes, how often have you worn hearing protection (earplugs, earmuffs)?				
	Never (or almost never)	Rarely	About half the time	Usually	Always (or almost always)
	0 ☐	1 ☐	2 ☐	3 ☐	4 ☐

A16	Do you know the cause of your hearing loss?		
	Yes	No	Not applicable
	1 ☐	2 ☐	3 ☐

→ **If No, go to question A18**

A17	**If yes, select the main causes:**
	1 □ Age-related hearing loss<br>2 □ Noise-induced hearing loss (e.g., Bang, Explosion, Shot, loud music)<br>3 □ Post surgical procedure<br>4 □ Ear disease (e.g., Infection, Otosclerosis, Injury, Acoustic neuroma, Meniere's disease, Cholesteatoma, etc.)<br>**Please specify:** ____________________<br>5 □ Accident, skull injury<br>6 □ Taking toxic medication, Ototoxicity<br>7 □ Congenital<br>8 □ Others; **please specify:** ____________________

A18	**Did you have a sudden hearing loss?**
	1 □ Yes<br><br>When?<br><br>What was the cause?<br>____________________<br>____________________<br>____________________<br>____________________<br><br>2 □ No<br>3 □ Not applicable<br>4 □ I don’t know

A19	**Have you had or will you have surgical treatments for ear and hearing conditions?**
	1 ☐ I previously had a surgery.<br><br>When?<br><br>In which ear? ☐ Right ☐ Left ☐ Both sides<br><br>What type of surgery was it?<br>____<br>____<br>____<br><br>2 ☐ A surgery is planned for the future.<br><br>Estimated date? ______________<br><br>Which ear? ☐ Right ☐ Left ☐ Both sides<br><br>What type of surgery will it be?<br>____<br>____<br>____<br><br>3 ☐ I have never had it and it’s not planned for the future.<br>4 ☐ I don't know

A20	**Have you ever been diagnosed with a middle ear infection?**
	1 ☐ Yes How old have you been?______________ (Year)<br>2 ☐ No<br>3 ☐ I don’t know

A21	**Do you suffer from runny ears (not normal ear wax, but abnormal moisture)?**		
	Yes	No	I don‘t know
	1 ☐	2 ☐	3 ☐

A22	**When was the last time you had your hearing tested?**					
	Never	Less than a year ago	**1** year to **4** years ago	**5** to **9** years ago	**10** or more years ago	I don't know
	0 ☐	1 ☐	2 ☐	3 ☐	4 ☐	5 ☐

A23	**Does anyone in your family also have an ear or hearing problem?**		
	Yes	No	I don't know
	1 ☐	2 ☐	3 ☐

→ If No, go to question A26

A24	**If yes, from which side of the family?**		
	Maternal	Paternal	Both
	1 ☐	2 ☐	3 ☐

A25	**If yes, please specify the relationship:**					
	Grandparents	Parents	Children	Siblings	Aunt or uncle	Cousins
	1 ☐	2 ☐	3 ☐	4 ☐	5 ☐	6 ☐

A26	**Does one of your ears hear better than the other?**			
	Most of the time	Only occasionally	No	I don’t know
	1 ☐	2 ☐	3 ☐	4 ☐

A26⁺	**Do you use headphones to enjoy TV or radio programs?**<br>**If yes, please also indicate the type of headphones and how often you use them!**
	1 □ No<br>2 □ Yes → If Yes, which type of headphones?<br>1 □ without cable (Wireless/Bluetooth/Infrared)<br>2 □ with cable<br><br>→ If Yes, how often?<br>1 □ one to several times a day<br>2 □ one to several times a day weekly<br>3 □ one to several times a day monthly<br>4 □ less frequently than once a month

A27	**Do you wear any hearing devices?**
	1 □ Yes (In which ear?) □ Right □ Left □ Both<br>2 □ No

If No, go to question H01

A28	**If yes, what type of hearing device do you wear?**
	Left ear:<br><br>1 ☐ Hearing aid (including Behind-The-Ear, In-The-Ear, Receiver In Canal)<br>2 ☐ Bone-Anchored Hearing Aid (e.g., BAHA, Ponto, Bonebridge, OSIA)<br>3 ☐ Middle Ear Implant (e.g., Tympanoplasty, Vibrant Soundbridge, Carina)<br>4 ☐ Cochlear Implant (CI)<br>5 ☐ Others; **please specify:** ____________________<br><br>Right ear:<br><br>1 ☐ Hearing aid (including Behind-The-Ear, In-The-Ear, Receiver In Canal)<br>2 ☐ Bone- Anchored Hearing Aid (e.g., BAHA, Ponto, Bonebridge, OSIA)<br>3 ☐ Middle Ear Implant (e.g., Tympanoplasty, Vibrant Soundbridge, Carina)<br>4 ☐ Cochlear Implant (CI)<br>5 ☐ Others; **please specify:** ____________________

A29	**If yes, for how long have you been using the hearing device? (In case you have a hearing aid in both ears, please specify it, separately)**
	Left device: since year ________<br>Right device: since year ________

A30	**If yes, how many hours a day do you use your hearing device?**			
	Less than **1** hour	**1** to **4** hours	**4** to **8** hours	More than **8** hours
	1 ☐	2 ☐	3 ☐	4 ☐

A30+	**Do you use add-on devices to stream content from TV sets, broadcast or telephone/ Video conference directly into your hearing aids ("streaming"?)**<br><br>**If yes, please specify the type of headphones and frequency of use!**
	1 ☐ No<br>2 ☐ Yes → If Yes, for what purpose? (Multiple answers)<br>1 ☐ TV set / Broadcast<br>2 ☐ Phone / Video conference<br><br>→ If Yes, how often do you use "streaming" for TV or Broadcast?<br>1 ☐ one to several times a day<br>2 ☐ one to several times a day weekly<br>3 ☐ one to several times a day monthly<br>4 ☐ less frequently than once a month<br><br>→ If Yes, how often do you use "streaming" for phone?<br>1 ☐ one to several times a day<br>2 ☐ one to several times a day weekly<br>3 ☐ one to several times a day monthly<br>4 ☐ less frequently than once a month

A30++	**Do you use additional devices to transmit content of conversations via an external microphone (e.g., Phonak "Roger", "mini mic", etc.) directly into your hearing aids ("streaming"?)**<br><br>**If yes, please indicate the frequency of use!**
	1 ☐ No<br>2 ☐ Yes → If Yes, how often do you use external microphones?<br>1 ☐ one to several times a day<br>2 ☐ one to several times a day weekly<br>3 ☐ one to several times a day monthly<br>4 ☐ less frequently than once a month

The following questions relate to your general everyday life and can also relate to hearing, but do not have to. We have put a broader focus here, which can also go beyond hearing. When answering the questions, think about the last 30 days considering both healthy and worse days. If you use hearing technologies such as a hearing aid or cochlear implant or other hearing devices, please answer the way you hear with them.

Functioning

H01	Do you have problems with mood (e.g., experience intense mood swings (shifts) and self-image issues, rapid issues in mood in a relatively short period of time)?						
	No problem / impairment	Mild problem / Impairment	Moderate problem / Impairment	Severe Problem / Impairment	Profound or Complete Problem / Impairment	I don't know	Not applicable
	0☐	1☐	2☐	3☐	4☐	5☐	6☐

H02	Do you have a problem with sleeping (falling asleep, waking up often during the night, or waking up early in the morning)?						
	No problem / impairment	Mild problem / Impairment	Moderate problem / Impairment	Severe Problem / Impairment	Profound or Complete Problem / Impairment	I don't know	Not applicable
	0☐	1☐	2☐	3☐	4☐	5☐	6☐

H03	Do you have a problem with focusing your attention on one thing?						
	No problem / impairment	Mild problem / Impairment	Moderate problem / Impairment	Severe Problem / Impairment	Profound or Complete Problem / Impairment	I don't know	Not applicable
	0☐	1☐	2☐	3☐	4☐	5☐	6☐

H04	Do you have a problem with maintaining your focus on two or more things at the same time?						
	No problem / impairment	Mild problem / Impairment	Moderate problem / Impairment	Severe Problem / Impairment	Profound or Complete Problem / Impairment	I don't know	Not applicable
	0☐	1☐	2☐	3☐	4☐	5☐	6☐

H05	Do you have a problem with remembering things?						
	No problem / impairment	Mild problem / Impairment	Moderate problem / Impairment	Severe Problem / Impairment	Profound or Complete Problem / Impairment	I don't know	Not applicable
	0☐	1☐	2☐	3☐	4☐	5☐	6☐

H06	Do you have a problem with recalling new information?						
	No problem / impairment	Mild problem / Impairment	Moderate problem / Impairment	Severe Problem / Impairment	Profound or Complete Problem / Impairment	I don't know	Not applicable
	0☐	1☐	2☐	3☐	4☐	5☐	6☐

H07	Do you have a problem with sad or depressed feelings?						
	No problem / impairment	Mild problem / Impairment	Moderate problem / Impairment	Severe Problem / Impairment	Profound or Complete Problem / Impairment	I don't know	Not applicable
	0☐	1☐	2☐	3☐	4☐	5☐	6☐

H08	Do you have a problem with seeing and recognizing a person you know across the road (with glasses or contact lenses, if necessary)?						
	No problem / impairment	Mild problem / Impairment	Moderate problem / Impairment	Severe Problem / Impairment	Profound or Complete Problem / Impairment	I don't know	Not applicable
	0☐	1☐	2☐	3☐	4☐	5☐	6☐

H09	Do you have a problem with seeing and recognizing an object at arm's length (with glasses or contact lenses, if necessary)?						
	No problem / impairment	Mild problem / Impairment	Moderate problem / Impairment	Severe Problem / Impairment	Profound or Complete Problem / Impairment	I don't know	Not applicable
	0☐	1☐	2☐	3☐	4☐	5☐	6☐

H10	**Do you have a problem with taste loss?**						
	No problem / impairment	Mild problem / Impairment	Moderate problem / Impairment	Severe Problem / Impairment	Profound or Complete Problem / Impairment	I don't know	Not applicable
	0☐	1☐	2☐	3☐	4☐	5☐	6☐

H11	**Do you have a problem with smell loss?**						
	No problem / impairment	Mild problem / Impairment	Moderate problem / Impairment	Severe Problem / Impairment	Profound or Complete Problem / Impairment	I don't know	Not applicable
	0☐	1☐	2☐	3☐	4☐	5☐	6☐

H12	**Do you have a problem with dizziness when standing or changing positions or walking or even when your head is still?**						
	No problem / impairment	Mild problem / Impairment	Moderate problem / Impairment	Severe Problem / Impairment	Profound or Complete Problem / Impairment	I don't know	Not applicable
	0☐	1☐	2☐	3☐	4☐	5☐	6☐

H13	**Do you have a problem with your balance when standing or walking or changing position (e.g., being unsteady or off-balance)?**						
	No problem / impairment	Mild problem / Impairment	Moderate problem / Impairment	Severe Problem / Impairment	Profound or Complete Problem / Impairment	I don't know	Not applicable
	0☐	1☐	2☐	3☐	4☐	5☐	6☐

H14	**Do you have a problem with pain in general?**						
	No problem / impairment	Mild problem / Impairment	Moderate problem / Impairment	Severe Problem / Impairment	Profound or Complete Problem / Impairment	I don't know	Not applicable
	0☐	1☐	2☐	3☐	4☐	5☐	6☐

H15	**Do you have a problem with pain in your head and neck area?**						
	No problem / impairment	Mild problem / Impairment	Moderate problem / Impairment	Severe Problem / Impairment	Profound or Complete Problem / Impairment	I don't know	Not applicable
	0☐	1☐	2☐	3☐	4☐	5☐	6☐
	**If you have a problem, please specify the exact area in which you feel the pain:**<br>**Which area?** ________________________________						

H16	Do you have a problem with understanding the meaning of a message in your language?						
	No problem / impairment	Mild problem / Impairment	Moderate problem / Impairment	Severe Problem / Impairment	Profound or Complete Problem / Impairment	I don't know	Not applicable
	0☐	1☐	2☐	3☐	4☐	5☐	6☐

H17	Do you have a problem with producing a meaningful message in your language?						
	No problem / impairment	Mild problem / Impairment	Moderate problem / Impairment	Severe Problem / Impairment	Profound or Complete Problem / Impairment	I don't know	Not applicable
	0☐	1☐	2☐	3☐	4☐	5☐	6☐

H18	Do you have a problem with ringing, beeping, roaring, or buzzing in your ears?						
	No problem / impairment	Mild problem / Impairment	Moderate problem / Impairment	Severe Problem / Impairment	Profound or Complete Problem / Impairment	I don't know	Not applicable
	0☐	1☐	2☐	3☐	4☐	5☐	6☐

H19	Do you have a problem with a feeling of pressure or pressure balance in your ear ("popping" of the ear) in your daily life?"						
	No problem / impairment	Mild problem / Impairment	Moderate problem / Impairment	Severe Problem / Impairment	Profound or Complete Problem / Impairment	I don't know	Not applicable
	0☐	1☐	2☐	3☐	4☐	5☐	6☐

H20	Do you have problems with irritation (e.g. itching) on or in the ear?						
	No problem / impairment	Mild problem / Impairment	Moderate problem / Impairment	Severe Problem / Impairment	Profound or Complete Problem / Impairment	I don't know	Not applicable
	0☐	1☐	2☐	3☐	4☐	5☐	6☐

H21	Do you have a problem with distinguishing the pitch of sounds in general?						
	No problem / impairment	Mild problem / Impairment	Moderate problem / Impairment	Severe Problem / Impairment	Profound or Complete Problem / Impairment	I don't know	Not applicable
	0☐	1☐	2☐	3☐	4☐	5☐	6☐

H22	Do you have a problem with distinguishing the tone of sounds in general?						
	No problem / impairment	Mild problem / Impairment	Moderate problem / Impairment	Severe Problem / Impairment	Profound or Complete Problem / Impairment	I don't know	Not applicable
	0☐	1☐	2☐	3☐	4☐	5☐	6☐

H23	Do you have a problem with distinguishing the volume of sounds in general?						
	No problem / impairment	Mild problem / Impairment	Moderate problem / Impairment	Severe Problem / Impairment	Profound or Complete Problem / Impairment	I don't know	Not applicable
	0☐	1☐	2☐	3☐	4☐	5☐	6☐

H24	Do you have a problem with detecting a sound in your surrounding environment in general?						
	No problem / impairment	Mild problem / Impairment	Moderate problem / Impairment	Severe Problem / Impairment	Profound or Complete Problem / Impairment	I don't know	Not applicable
	0☐	1☐	2☐	3☐	4☐	5☐	6☐

H25	Do you have a problem with detecting noises in the household, like running water or a washing machine?						
	No problem / impairment	Mild problem / Impairment	Moderate problem / Impairment	Severe Problem / Impairment	Profound or Complete Problem / Impairment	I don't know	Not applicable
	0☐	1☐	2☐	3☐	4☐	5☐	6☐

H26	Do you have a problem with discriminating between the sound of a car and a bus?						
	No problem / impairment	Mild problem / Impairment	Moderate problem / Impairment	Severe Problem / Impairment	Profound or Complete Problem / Impairment	I don't know	Not applicable
	0☐	1☐	2☐	3☐	4☐	5☐	6☐

H27	Do you have a problem with recognizing which instruments are playing when you are listening to music?						
	No problem / impairment	Mild problem / Impairment	Moderate problem / Impairment	Severe Problem / Impairment	Profound or Complete Problem / Impairment	I don't know	Not applicable
	0☐	1☐	2☐	3☐	4☐	5☐	6☐

H28	Do you have a problem with detecting where a sound comes from?						
	No problem / impairment	Mild problem / Impairment	Moderate problem / Impairment	Severe Problem / Impairment	Profound or Complete Problem / Impairment	I don't know	Not applicable
	0☐	1☐	2☐	3☐	4☐	5☐	6☐

H29	Do you have a problem with telling whether a bus or truck is coming towards you or going away?						
	No problem / impairment	Mild problem / Impairment	Moderate problem / Impairment	Severe Problem / Impairment	Profound or Complete Problem / Impairment	I don't know	Not applicable
	0☐	1☐	2☐	3☐	4☐	5☐	6☐

H30	Do you have a problem with detecting from what corner of a lecture room someone is asking a question during a meeting?						
	No problem / impairment	Mild problem / Impairment	Moderate problem / Impairment	Severe Problem / Impairment	Profound or Complete Problem / Impairment	I don't know	Not applicable
	0☐	1☐	2☐	3☐	4☐	5☐	6☐

H31	Do you have a problem with telling how far away a bus or a truck is from the sound?						
	No problem / impairment	Mild problem / Impairment	Moderate problem / Impairment	Severe Problem / Impairment	Profound or Complete Problem / Impairment	I don't know	Not applicable
	0☐	1☐	2☐	3☐	4☐	5☐	6☐

H32	Do you have a problem with telling where a human is when he screams or where a dog is when it barks loudly without having to look?						
	No problem / impairment	Mild problem / Impairment	Moderate problem / Impairment	Severe Problem / Impairment	Profound or Complete Problem / Impairment	I don't know	Not applicable
	0☐	1☐	2☐	3☐	4☐	5☐	6☐

H33	Do you have a problem with detecting right away whether the person on your left or the person on your right starts talking, without having to look?						
	No problem / impairment	Mild problem / Impairment	Moderate problem / Impairment	Severe Problem / Impairment	Profound or Complete Problem / Impairment	I don't know	Not applicable
	0☐	1☐	2☐	3☐	4☐	5☐	6☐

H34	**Do you have a problem with hearing a single jumbled sound when you are hearing more than one sound at a time?**						
	No problem / impairment	Mild problem / Impairment	Moderate problem / Impairment	Severe Problem / Impairment	Profound or Complete Problem / Impairment	I don’t know	Not applicable
	0□	1□	2□	3□	4□	5□	6□

H35	**Do you have a problem with understanding the speech of someone you know (your close family members and friends) over a distance of two or more meters?**						
	No problem / impairment	Mild problem / Impairment	Moderate problem / Impairment	Severe Problem / Impairment	Profound or Complete Problem / Impairment	I don’t know	Not applicable
	0□	1□	2□	3□	4□	5□	6□

H36	**Do you have a problem with understanding the speech of someone you know (your close family members and friend) in a quiet environment?**						
	No problem / impairment	Mild problem / Impairment	Moderate problem / Impairment	Severe Problem / Impairment	Profound or Complete Problem / Impairment	I don’t know	Not applicable
	0□	1□	2□	3□	4□	5□	6□

H37	**Do you have a problem with understanding the speech of someone you know (your close family members and friend) in a noisy environment?**						
	No problem / impairment	Mild problem / Impairment	Moderate problem / Impairment	Severe Problem / Impairment	Profound or Complete Problem / Impairment	I don’t know	Not applicable
	0□	1□	2□	3□	4□	5□	6□

H38	**Do you have a problem with understanding the presenter of the news on the radio or TV in general?**						
	No problem / impairment	Mild problem / Impairment	Moderate problem / Impairment	Severe Problem / Impairment	Profound or Complete Problem / Impairment	I don’t know	Not applicable
	0□	1□	2□	3□	4□	5□	6□

H39	**Do you have a problem with understanding what someone is saying while the TV is on at the same time without turning the TV down?**						
	No problem / impairment	Mild problem / Impairment	Moderate problem / Impairment	Severe Problem / Impairment	Profound or Complete Problem / Impairment	I don’t know	Not applicable
	0□	1□	2□	3□	4□	5□	6□

H40	Do you have a problem with understanding the presenter of the news on the radio or TV and understanding what someone is saying at the same time?					
No problem / impairment	Mild problem / Impairment	Moderate problem / Impairment	Severe Problem / Impairment	Profound or Complete Problem / Impairment	I don't know	Not applicable
0☐	1☐	2☐	3☐	4☐	5☐	6☐

H41	Do you have any health conditions causing speech impairment or producing sounds? (e.g., caused by ENT problems, stroke, head injury, and other diseases?	
Yes	No	I don't know
0☐	1☐	2☐

→ If No, go to question H49

H42	If yes, have you been told by other people that you have problems with making sounds (other than speech) such as whistling with your mouth? How big was the problem from the other person's point of view?					
No problem / impairment	Mild problem / Impairment	Moderate problem / Impairment	Severe Problem / Impairment	Profound or Complete Problem / Impairment	I don't know	Not applicable
0☐	1☐	2☐	3☐	4☐	5☐	6☐

H43	If yes, have you been told by others that you have problems with changing the pitch of sounds (other than speech), such as whistles? How big was the problem from the other person's point of view?					
No problem / impairment	Mild problem / Impairment	Moderate problem / Impairment	Severe Problem / Impairment	Profound or Complete Problem / Impairment	I don't know	Not applicable
0☐	1☐	2☐	3☐	4☐	5☐	6☐

H44	If yes, have you been told by others that you have problems with changing the volume of sounds (other than speech), such as whistling? How big was the problem from the other person's point of view?					
No problem / impairment	Mild problem / Impairment	Moderate problem / Impairment	Severe Problem / Impairment	Profound or Complete Problem / Impairment	I don't know	Not applicable
0☐	1☐	2☐	3☐	4☐	5☐	6☐

H45	If yes, have you been told by other people that you have problems with pronunciation? How big was the problem from the other person's point of view?					
No problem / impairment	Mild problem / Impairment	Moderate problem / Impairment	Severe Problem / Impairment	Profound or Complete Problem / Impairment	I don't know	Not applicable
0☐	1☐	2☐	3☐	4☐	5☐	6☐

H46	If yes, have you been told by other people that you have problems with changing the volume of your speech (too soft or too loud)? How big was the problem from the other person's point of view?						
	No problem / impairment	Mild problem / Impairment	Moderate problem / Impairment	Severe Problem / Impairment	Profound or Complete Problem / Impairment	I don't know	Not applicable
	0☐	1☐	2☐	3☐	4☐	5☐	6☐

H47	If yes, have you been told by other people that you have problems with changing the speed of your speech? How big was the problem from the other person's point of view?						
	No problem / impairment	Mild problem / Impairment	Moderate problem / Impairment	Severe Problem / Impairment	Profound or Complete Problem / Impairment	I don't know	Not applicable
	0☐	1☐	2☐	3☐	4☐	5☐	6☐

H48	If yes, have you been told by other people that you have problems with telling stories or reporting on something? How big was the problem from the other person's point of view?						
	No problem / impairment	Mild problem / Impairment	Moderate problem / Impairment	Severe Problem / Impairment	Profound or Complete Problem / Impairment	I don't know	Not applicable
	0☐	1☐	2☐	3☐	4☐	5☐	6☐

Activity Limitations and Participation Restrictions

H49	Do you have difficulty with dealing with stressful situations?						
	No difficulty	Mild difficulty	Moderate difficulty	Severe difficulty	Profound / Complete difficulty	I don't know	Not applicable
	0☐	1☐	2☐	3☐	4☐	5☐	6☐

H50	Do you have difficulty with interacting with people in a socially appropriate manner (e.g. regulating emotions, controlling verbal and physical aggression)?						
	No difficulty	Mild difficulty	Moderate difficulty	Severe difficulty	Profound / Complete difficulty	I don't know	Not applicable
	0☐	1☐	2☐	3☐	4☐	5☐	6☐

H51	Do you have difficulty with socializing with people living in your community (e.g. classmates, co-workers)?						
	No difficulty	Mild difficulty	Moderate difficulty	Severe difficulty	Profound / Complete difficulty	I don't know	Not applicable
	0☐	1☐	2☐	3☐	4☐	5☐	6☐

H52	Do you have difficulty with dealing with people you do not know?						
	No difficulty	Mild difficulty	Moderate difficulty	Severe difficulty	Profound / Complete difficulty	I don't know	Not applicable
	0☐	1☐	2☐	3☐	4☐	5☐	6☐

H53	Do you have difficulty with starting and continuing formal relationships with people in authority (e.g. employers, professionals, or service providers)?						
	No difficulty	Mild difficulty	Moderate difficulty	Severe difficulty	Profound / Complete difficulty	I don't know	Not applicable
	0☐	1☐	2☐	3☐	4☐	5☐	6☐

H54	Do you have difficulty with socializing with your family or friends?						
	No difficulty	Mild difficulty	Moderate difficulty	Severe difficulty	Profound / Complete difficulty	I don't know	Not applicable
	0☐	1☐	2☐	3☐	4☐	5☐	6☐

H55	Do you have difficulty with making new friends?						
	No difficulty	Mild difficulty	Moderate difficulty	Severe difficulty	Profound / Complete difficulty	I don't know	Not applicable
	0☐	1☐	2☐	3☐	4☐	5☐	6☐

H56	Do you have difficulty with starting, continuing, or ending an argument or debate with one person or many people?						
	No difficulty	Mild difficulty	Moderate difficulty	Severe difficulty	Profound / Complete difficulty	I don't know	Not applicable
	0☐	1☐	2☐	3☐	4☐	5☐	6☐

H57	Do you have difficulty with understanding a statement or question during communication activity?					
No difficulty	Mild difficulty	Moderate difficulty	Severe difficulty	Profound / Complete difficulty	I don't know	Not applicable
0☐	1☐	2☐	3☐	4☐	5☐	6☐

H58	Do you have difficulty with maintaining relationships with your immediate family members (Parents, partner, children)?					
No difficulty	Mild difficulty	Moderate difficulty	Severe difficulty	Profound / Complete difficulty	I don't know	Not applicable
0☐	1☐	2☐	3☐	4☐	5☐	6☐

H59	Do you have difficulty with joining in community activities (for example, festivities, religious or other activities) in the same way as anyone else can?					
No difficulty	Mild difficulty	Moderate difficulty	Severe difficulty	Profound / Complete difficulty	I don't know	Not applicable
0☐	1☐	2☐	3☐	4☐	5☐	6☐

H60	Do you have difficulty with engaging in any hobby or pleasurable activity (such as games, sports, or going to the cinema)?					
No difficulty	Mild difficulty	Moderate difficulty	Severe difficulty	Profound / Complete difficulty	I don't know	Not applicable
0☐	1☐	2☐	3☐	4☐	5☐	6☐

H61	Do you have difficulty with starting and continuing relationships in a socially appropriate manner (e.g. regulating emotions, controlling verbal and physical aggression)?					
No difficulty	Mild difficulty	Moderate difficulty	Severe difficulty	Profound / Complete difficulty	I don't know	Not applicable
0☐	1☐	2☐	3☐	4☐	5☐	6☐

H62	Do you have difficulty with performing communication techniques such as lip-reading?					
No difficulty	Mild difficulty	Moderate difficulty	Severe difficulty	Profound / Complete difficulty	I don't know	Not applicable
0☐	1☐	2☐	3☐	4☐	5☐	6☐

Note: Answer questions H63 to H66 with regards to the task you are assigned at your school or university, paid or unpaid work.

H63	Do you have difficulty with your day-to-day occupation/tasks?						
	No difficulty	Mild difficulty	Moderate difficulty	Severe difficulty	Profound / Complete difficulty	I don't know	Not applicable
	0☐	1☐	2☐	3☐	4☐	5☐	6☐

H64	Do you have difficulty with doing your most important tasks well?						
	No difficulty	Mild difficulty	Moderate difficulty	Severe difficulty	Profound / Complete difficulty	I don't know	Not applicable
	0☐	1☐	2☐	3☐	4☐	5☐	6☐

H65	Do you have difficulty with getting done all the tasks that you needed to do?						
	No difficulty	Mild difficulty	Moderate difficulty	Severe difficulty	Profound / Complete difficulty	I don't know	Not applicable
	0☐	1☐	2☐	3☐	4☐	5☐	6☐

H66	Do you have difficulty with getting your tasks done as quickly as needed?						
	No difficulty	Mild difficulty	Moderate difficulty	Severe difficulty	Profound / Complete difficulty	I don't know	Not applicable
	0☐	1☐	2☐	3☐	4☐	5☐	6☐

H67	Do you have difficulty with starting, continuing, or ending a conversation, or speaking with someone?						
	No difficulty	Mild difficulty	Moderate difficulty	Severe difficulty	Profound / Complete difficulty	I don't know	Not applicable
	0☐	1☐	2☐	3☐	4☐	5☐	6☐

H68	Do you have difficulty with starting, continuing, or ending a conversation, or speaking with several people in a group?						
	No difficulty	Mild difficulty	Moderate difficulty	Severe difficulty	Profound / Complete difficulty	I don't know	Not applicable
	0☐	1☐	2☐	3☐	4☐	5☐	6☐

H69	Do you have difficulty with carrying on a conversation with someone during a crowded meeting?						
	No difficulty	Mild difficulty	Moderate difficulty	Severe difficulty	Profound / Complete difficulty	I don't know	Not applicable
	0☐	1☐	2☐	3☐	4☐	5☐	6☐

H70	Do you have difficulty with carrying on a conversation with somebody in a bus or car? Think about the transportation you use on daily basis.						
	No difficulty	Mild difficulty	Moderate difficulty	Severe difficulty	Profound / Complete difficulty	I don't know	Not applicable
	0☐	1☐	2☐	3☐	4☐	5☐	6☐

H71	Do you have difficulty with following a conversation between five people in a busy restaurant while you can see everyone?						
	No difficulty	Mild difficulty	Moderate difficulty	Severe difficulty	Profound / Complete difficulty	I don't know	Not applicable
	0☐	1☐	2☐	3☐	4☐	5☐	6☐

H72	Do you have difficulty with carrying a phone call in a quiet room?						
	No difficulty	Mild difficulty	Moderate difficulty	Severe difficulty	Profound / Complete difficulty	I don't know	Not applicable
	0☐	1☐	2☐	3☐	4☐	5☐	6☐

H73	Do you have difficulty with telling what someone is saying when the conversation switches from one person to another?						
	No difficulty	Mild difficulty	Moderate difficulty	Severe difficulty	Profound / Complete difficulty	I don't know	Not applicable
	0☐	1☐	2☐	3☐	4☐	5☐	6☐

H74	Do you have difficulty with listening to the television, radio, or music in general?						
	No difficulty	Mild difficulty	Moderate difficulty	Severe difficulty	Profound / Complete difficulty	I don't know	Not applicable
	0☐	1☐	2☐	3☐	4☐	5☐	6☐

Environmental factors

H75	What is the extent to which you rate the general support received from people in your society (such as providing emotional and social support, encouragement, etc.)?					
No support	Mild support	Moderate support	Substantial support	Complete support	I don't know	Not applicable
0☐	1☐	2☐	3☐	4☐	5☐	6☐

H76	What is the extent to which you rate the general support received from your close family members and friends (such as providing emotional and social support, encouragement, and so on)?					
No support	Mild support	Moderate support	Substantial support	Complete support	I don't know	Not applicable
0☐	1☐	2☐	3☐	4☐	5☐	6☐

H77	What is the extent to which you rate the general support received from your close family members and friends in your daily functioning, especially during listening-conversation activities?					
No support	Mild support	Moderate support	Substantial support	Complete support	I don't know	Not applicable
0☐	1☐	2☐	3☐	4☐	5☐	6☐

H78	What is the extent to which you rate the general support received from the main health services and systems offered in relation to your hearing aids and medical services (e.g. ear specialist)?					
No support	Mild support	Moderate support	Substantial support	Complete support	I don't know	Not applicable
0☐	1☐	2☐	3☐	4☐	5☐	6☐

H79	What is the extent to which you rate the general support received from your most important healthcare professional(s)?					
No support	Mild support	Moderate support	Substantial support	Complete support	I don't know	Not applicable
0☐	1☐	2☐	3☐	4☐	5☐	6☐

H80	What is the extent to which you rate the overall usefulness of the communication services and systems you use daily such as telephone, cellphone, speaker, Bluetooth connection, and so on?					
No usefulness	Mild usefulness	Moderate usefulness	Substantial usefulness	Complete usefulness	I don't know	Not applicable
0☐	1☐	2☐	3☐	4☐	5☐	6☐

Note: When answering questions H81 to H86, think of a barrier as a hindrance, added difficulty, and restriction. Answer these questions considering the barrier that can affect your daily functioning/tasks (e.g., during listening-conversation activities).

**H81**	**What is the extent to which the design and construction of your workplace/task place can be considered a barrier? Think about video conferencing, as an example!**						
	No barrier	Mild barrier	Moderate barrier	Severe barrier	Complete barrier	I don't know	Not applicable
	0☐	1☐	2☐	3☐	4☐	5☐	6☐

**H82**	**What is the extent to which the darkness or insufficient light can be considered a barrier (e.g., in lip-reading)?**						
	No barrier	Mild barrier	Moderate barrier	Severe barrier	Complete barrier	I don't know	Not applicable
	0☐	1☐	2☐	3☐	4☐	5☐	6☐

**H83**	**What is the extent to which the low volume of speech can be considered a barrier?**						
	No barrier	Mild barrier	Moderate barrier	Severe barrier	Complete barrier	I don't know	Not applicable
	0☐	1☐	2☐	3☐	4☐	5☐	6☐

**H84**	**What is the extent to which the background noise can be considered a barrier?**						
	No barrier	Mild barrier	Moderate barrier	Severe barrier	Complete barrier	I don't know	Not applicable
	0☐	1☐	2☐	3☐	4☐	5☐	6☐

**H85**	**What is the extent to which the reverberant or echoing environment (e.g., train station) can be considered a barrier?**						
	No barrier	Mild barrier	Moderate barrier	Severe barrier	Complete barrier	I don't know	Not applicable
	0☐	1☐	2☐	3☐	4☐	5☐	6☐

**H86**	**What is the extent to which the unclear sound can be considered a barrier?**						
	No barrier	Mild barrier	Moderate barrier	Severe barrier	Complete barrier	I don't know	Not applicable
	0☐	1☐	2☐	3☐	4☐	5☐	6☐

Please answer questions H87 to H90 only if you have hearing aids!

H87	What is the extent to which you rate the overall usefulness of your hearing aid in your normal daily routines?						
	No usefulness	Mild usefulness	Moderate usefulness	Substantial usefulness	Complete usefulness	I don't know	Not applicable
	0☐	1☐	2☐	3☐	4☐	5☐	6☐

H88	What is the extent to which you rate the overall usefulness of your hearing aid during listening-conversation activities?						
	No usefulness	Mild usefulness	Moderate usefulness	Substantial usefulness	Complete usefulness	I don't know	Not applicable
	0☐	1☐	2☐	3☐	4☐	5☐	6☐

H89	What is the extent to which you rate the overall usefulness of your hearing aid while using a telephone or cellphone?						
	No usefulness	Mild usefulness	Moderate usefulness	Substantial usefulness	Complete usefulness	I don't know	Not applicable
	0☐	1☐	2☐	3☐	4☐	5☐	6☐

H90	What is the extent to which you rate the overall usefulness of your hearing aid while watching TV?						
	No usefulness	Mild usefulness	Moderate usefulness	Substantial usefulness	Complete usefulness	I don't know	Not applicable
	0☐	1☐	2☐	3☐	4☐	5☐	6☐

**Free comments**<br>**Please indicate here any other aspects that we may not have asked about, e.g., barriers and obstacles in everyday life that are relevant to hearing or other aspects that are important to you.**

Thank you for your collaboration.
